# Cohort Profile: The Cohorts Consortium of Latin America and the Caribbean (CC-LAC)

**DOI:** 10.1093/ije/dyaa073

**Published:** 2020-09-05

**Authors:** Cohorts Consortium of Latin America and the Caribbean (CC-LAC), Rodrigo M Carrillo-Larco, Mariachiara Di Cesare, Ian R Hambleton, Anselm Hennis, Vilma Irazola, Dalia Stern, Catterina Ferreccio, Paulo Lotufo, Pablo Perel, Edward W Gregg, Majid Ezzati, Goodarz Danaei, J Jaime Miranda, Carlos A Aguilar-Salinas, Ramón Alvarez-Váz, Marselle B Amadio, Cecilia Baccino, Claudia Bambs, João Luiz Bastos, Gloria Beckles, Antonio Bernabe-Ortiz, Carla D O Bernardo, Katia V Bloch, Juan E Blümel, Jose G Boggia, Pollyanna K Borges, Miguel Bravo, Gilbert Brenes-Camacho, Horacio A Carbajal, Maria S Castillo Rascon, Blanca H Ceballos, Veronica Colpani, Susana C Confortin, Jackie A Cooper, Adrian Cortés-Valencia, Sandra Cortes, Roberto S Cunha, Eleonora d'Orsi, William H Dow, Walter G Espeche, Flavio D Fuchs, Sandra C Fuchs, Suely G A Gimeno, Donaji Gomez-Velasco, Clicerio Gonzalez-Villalpando, María-Elena Gonzalez-Villalpando, David A Gonzalez-Chica, Gonzalo Grazioli, Ricardo O Guerra, Laura Gutierrez, Fernando L Herkenhoff, Andrea R V R Horimoto, Andrea Huidobro, Elard Koch, Martin Lajous, Maria Fernanda Lima-Costa, Ruy Lopez-Ridaura, Alvaro C C Maciel, Betty S Manrique-Espinoza, Larissa P Marques, Jose G Mill, Leila B Moreira, Lariane M Ono, Oscar M Muñoz, Karen Oppermann, Sergio V Peixoto, Alexandre C Pereira, Karen G Peres, Marco A Peres, Nohora I Rodriguez, Rosalba Rojas-Martinez, Luis Rosero-Bixby, Adolfo Rubinstein, Alvaro Ruiz-Morales, Martin R Salazar, Aaron Salinas-Rodriguez, Ramon A Sanchez, Ione J C Schneider, Thiago L N Silva, Nelson A S Silva, Liam Smeeth, Poli M Spritzer, Fiorella Tartaglione, Jorge Tartaglione

## Why was the cohort set up?

Latin America and the Caribbean (LAC) are characterized by much diversity in terms of socio-economic status, ecology, environment, access to health care,^1^^,^^2^ as well as the frequency of risk factors for and prevalence or incidence of non-communicable diseases;^3–7^ importantly, these differences are observed both between and within countries in LAC.^8^^,^^9^ LAC countries share a large burden of non-communicable (e.g. diabetes and hypertension) and cardiovascular (e.g. ischaemic heart disease) diseases, with these conditions standing as the leading causes of morbidity, disability and mortality in most of LAC.^10–12^ These epidemiological estimates—e.g. morbidity—cannot inform about risk factors or risk prediction, which are relevant to identify prevention avenues. Cohort studies, on the other hand, could provide this evidence. Pooled analysis, using data from multiple cohort studies, have additional strengths such as increased statistical power and decreased statistical uncertainty.^13^ LAC cohort studies have been under-represented,^14^ or not included at all,^15–17^ in international efforts aimed at pooling data from multiple cohort studies. We therefore set out to pool data from LAC cohorts to address research questions that individual cohort studies would not be able to answer.

Drawing from previous successful regional enterprises (e.g. Asia Pacific Cohort Studies Collaboration),^18^^,^^19^ we established the Cohorts Consortium of Latin America and the Caribbean (CC-LAC). The main aim of the CC-LAC is to start a collaborative cohort data pooling in LAC to examine the association between cardio-metabolic risk factors (e.g. blood pressure, glucose and lipids) and non-fatal and fatal cardiovascular outcomes (e.g. stroke or myocardial infarction). In so doing, we aim to provide regional risk estimates to inform disease burden metrics, as well as other ambitious projects including a cardiovascular risk score to strengthen cardiovascular prevention in LAC.

Initial funding has been provided by a fellowship from the Wellcome Trust Centre for Global Health Research at Imperial College London (Strategic Award, Wellcome Trust–Imperial College Centre for Global Health Research, 100693/Z/12/Z). Additional funding is being provided by an International Training Fellowship from the Wellcome Trust (214185/Z/18/Z). At the time of writing, the daily operations and pooled database are hosted at Imperial College London, though a mid-term goal is to transfer this expertise and operations to LAC. The collaboration relies fundamentally on a strong regional network of health researchers and practitioners.

## Who is in the cohort?

We have harmonized and pooled approximately population-based cohort data on cardio-metabolic risk factors and outcomes, i.e. participants were not recruited based on disease (e.g. cohort of stroke survivors) or risk factor (e.g. cohort of smokers) history. Data have been collated by the CC-LAC, a LAC network of health researchers and practitioners. The database was collated using multiple data identification sources. First, we accessed publicly available cohort data through each study’s website or data repository. Second, we conducted a systematic search in Medline, Embase and SciELO, a LAC-based search engine to identify cohort studies with peer-reviewed publications in regional journals. The search query included country terms (countries in LAC), cohort studies (e.g. cohort stud*), and cardiovascular outcomes (e.g. stroke); the search query is available in Supplementary data p. 02, available as Supplementary data at *IJE* online. We invited all eligible cohorts to join the CC-LAC requesting access to anonymized individual-level data. Third, enquiries among LAC researchers also helped identify or approach principal investigators of the identified cohorts. LAC members of the Non-Communicable Disease Risk Factor Collaboration (NCD-RisC)^20^ also helped identify additional data sources. Sources that led to the identification of the collaborating cohort studies are shown in Supplementary data pp. 03–06, available as Supplementary data at *IJE* online. The project procedures were approved by the Institutional Review Board at Universidad Peruana Cayetano Heredia (UPCH), Lima, Peru.

Data sources were included in the CC-LAC database if they had at least a baseline and one follow-up round, included LAC populations living in LAC, and participants were not recruited exclusively based on the presence of a risk factor or disease. No restrictions were set regarding a minimum sample size at baseline or follow-up duration, sex or age profiles. Anonymized individual record data were received, harmonized and pooled by the CC-LAC. During the harmonization process, we excluded participants who by the time of the baseline assessment had had a cardiovascular event (e.g. stroke or myocardial infarction). Implausible values in selected risk factors were excluded as follows:^5–7^^,^^21^ body mass index (BMI <10 kg/m^2^ or >80 kg/m^2^), systolic blood pressure (<70 mmHg or >270 mmHg), diastolic blood pressure (<30 mmHg or >150 mmHg), total cholesterol (<1.75 mmol/L or >20.00 mmol/L), high-density lipoprotein (HDL)-cholesterol (<0.40 mmol/L or >5.00 mmol/L), and fasting glucose (<2.50 mmol/L or >30.00 mmol/L) (Supplementary data are available at *IJE* online p. 07). Notably, BMI was computed based on measured weight and height in all but one Mexican cohort,^22^ yet the self-reported weight and height closely correlated with measured estimates.^23^

**Figure 1:**
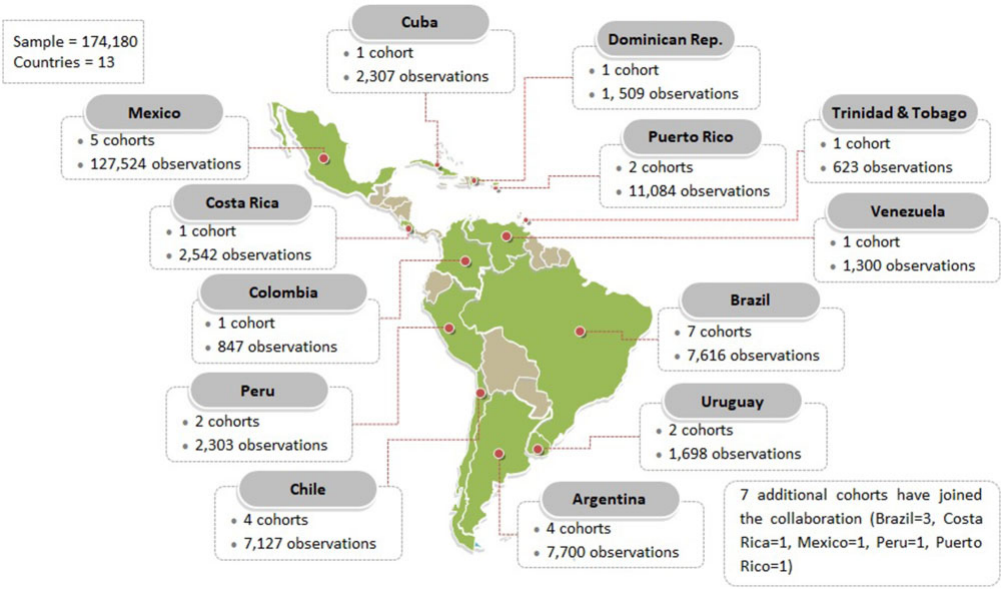
Number of pooled cohorts and participants by country in Latin America and the Caribbean. Based on the 32 pooled cohort units (total sample size = 174 180). A unit is a cohort-country, i.e. cohorts in multiple countries are counted as many times as countries were included (e.g., CESCAS-Argentina, CESCAS-Chile and CESCAS-Uruguay)^24^.

At the time of writing, i.e. first datalock on 29 March 2020, 32 cohorts with data on cardiovascular outcomes have been pooled. Seven additional cohorts have joined the CC-LAC but are currently working to provide cardiovascular outcomes, thus these 7 cohorts have not been pooled yet. To date, the CC-LAC is therefore a consortium of 39 cohorts in 13 LAC countries (Figure 1).

Among those cohorts we currently have pooled, there are two multi-country studies: Centro de Excelencia en Salud Cardiovascular para América del Sur (CESCAS) Cohort (Argentina, Chile and Uruguay)^24^ and the 10/66 Study (Cuba, Dominican Republic, Peru, Venezuela, Mexico and Puerto Rico),^25^^,^^26^ whereas the other cohort studies were based in only one country. The largest sample size is with The Mexican Teachers’ Cohort (Table 2).^22^ A list of all pooled cohorts along with supporting references is presented in Supplementary data pp. 03–06, available as Supplementary data at *IJE* online. 

**Table 2.** Available cardio-metabolic risk factors across collaborating cohorts; further details about available variables at follow-up are presented in Supplementary data pp. 17–18, available as Supplementary data at *IJE* online

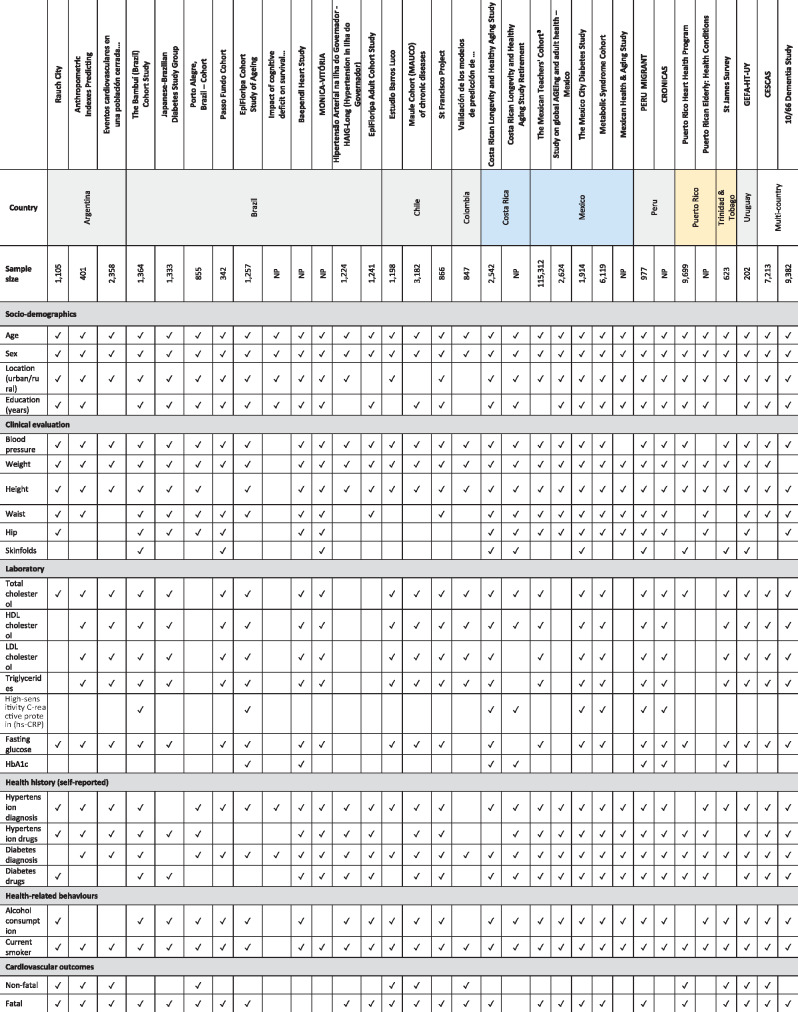

a Self-reported height/weight.

CESCAS, Centro de Excelencia en Salud Cardiovascular para América del Sur; GEFA-HT-UY, GEnotipo, Fenotipo y Ambiente de la HiperTensión Arterial en UruguaY; NP, not pooled yet.

The aforementioned 7 cohorts that have not been pooled because cardiovascular outcomes are not yet available include: CRONICAS Cohort Study (Peru),^27^ Mexican Health & Aging Study (Mexico),^28^ Puerto Rican Elderly Health Conditions (Puerto Rico),^29^ Costa Rican Longevity and Healthy Aging Study (CRELES) 1945–1955 Retirement Cohort (Costa Rica),^30^ Influence of Biopsychosocial Factors on the Survival of the Elderly in Northeast Brazil—A Prospective Study (Brazil),^31^ Baependi Heart Study (Brazil)^32^ and MONICA-VITÓRIA study (Brazil).^33^

The pooled dataset including all cohorts is not designed to be fully representative of the populations of regions and countries in which they have been conducted. Nevertheless, the results provided by this consortium will be informative for most of the LAC region, where large, longitudinal and multi-country studies remain insufficient in the field of cardiovascular diseases risk prediction. This consortium aims to advance the regional scientific evidence by overcoming the limitations of individual cohorts, e.g. studies with small sample size or limited number of events, while building upon a strong collaborative network of investigators.

## How often have they been followed up?

The CC-LAC has pooled the baseline assessment and the latest follow-up available for each cohort; for the purpose of this consortium all subjects have been followed once, i.e. at baseline and one follow-up. All pooled individuals have the outcomes of interest, either non-fatal or fatal cardiovascular events (or censored). At the time of the first datalock, the 32 pooled cohorts had a mean follow-up time to the first cardiovascular non-fatal/fatal event of 8.50 (median = 8.80) years, ranging from <1.0 to 27.7 years.

**Figure 2:**
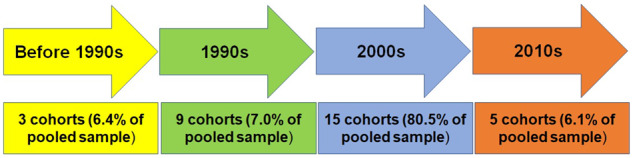
Number of pooled cohorts and sample size percentage according to when the baseline assessment was conducted. Based on the 32 cohorts that have been pooled. Total sample size of 174 180.

Figure 2 shows the number of cohort studies and the percentage of the pooled sample size at baseline. Most pooled cohorts started in the 2000s. Furthermore, 15.7% (*n* = 27 409) of the pooled sample had <5 years of follow-up, 59.3% (*n* = 103 254) between 5 and 9 years and 25.0% (*n* = 43 517) >10 years of follow-up.

## What has been measured?

Table 1 shows the number of observations per pooled cardio-metabolic risk factor. In addition to these variables, some cohorts have collected additional anthropometric measurements (e.g. waist/hip circumference or skinfold thickness) and laboratory data (e.g. Glycated hemoglobin (HbA1c), triglycerides, low-density lipoprotein (LDL)-cholesterol). Table 2 depicts the cardio-metabolic risk factors available across the pooled cohorts, and Supplementary data pp. 17–18, available as Supplementary data at *IJE* online, show further information on these variables at follow-up rounds. In addition to clinical cardio-metabolic risk factors, we have pooled two relevant socio-demographic indicators: place of residence (urban or rural) and schooling (years of education). 

**Table 1.** Number of available observations per pooled variable of interest, based on 32 pooled cohorts including 174 180 individuals

Variable	Sample size
Sex (male and female)	29 474 and 144 694
Body mass index (kg/m^2^)	152 412
Total cholesterol (mg/dL)	54 255
HDL-cholesterol (mg/dL)	39 566
Fasting glucose (mg/dL)	53 518
Systolic blood pressure (mmHg)	63 066
Current smoker	166 904
Location (urban/rural)	168 892
Education (none, <12 years, 12+ years)	115 485
Complete follow-up/censored	171 937
First non-fatal event	578
Fatal event	1665
Mean follow-up time (years)	8.50 (standard deviation = 3.27)

Two outcomes of interest have been harmonized and pooled, these are non-fatal and fatal cardiovascular events: haemorrhagic and ischaemic stroke, myocardial infarction (including revascularization), and mortality due to these conditions, as well as sudden death. Where relevant, these outcomes have been adjudicated or extracted from reliable sources, such as clinical records or death registries. Details on the ascertainment methods of these outcomes are provided in Supplementary data pp. 8–16, available as Supplementary data at *IJE* online.

At the time of the first datalock, data from 174 180 individuals have been pooled: 171 937 (98.7%) have complete follow-up or were censored (e.g. lost-to follow-up or death by other causes), 578 (0.3%) have had a first non-fatal cardiovascular event and 1665 (1.0%) have experienced a fatal cardiovascular event (Table 1).

## What has it found? Key findings and publications

This is the first of a series of anticipated outputs and therefore constitutes a key initial publication to provide a general overview of this regional long-needed endeavour. This cohort profile aims to inform the international research and clinical community about our ongoing efforts and inform them of our anticipated forthcoming outcomes. Our ongoing work includes: (i) age-specific risk estimates of cardio-metabolic risk factors on cardiovascular non-fatal and fatal outcomes; (ii) comparative risk assessment of current versus ideal levels of cardio-metabolic risk factors based on regional age-specific risk estimates and survey data; and (iii) a novel risk score for cardiovascular events based on LAC hazard estimates, and country-specific re-calibration with LAC population-based estimates of risk factors. Although other relevant efforts have provided cardiovascular risk charts for LAC,^34^^,^^35^ they all relied on hazard estimates from cohorts outside LAC which may not accurately apply to LAC populations.

Besides scientific publications and other relevant outputs, CC-LAC has found that several cohort studies have been conducted in LAC and provide strong data to advance the knowledge of cardiovascular health in LAC. Probably, these cohort studies were not included in previous international cohort data pooling efforts because of language barriers or because LAC cohorts are rather young, i.e. started mostly in the 2000s. Moreover, CC-LAC has demonstrated that it is feasible to conduct a large-scale cohort data pooling effort across LAC. We encourage other research groups working in different fields across health sciences (e.g. infectious diseases, climate change and maternal/child health) to also embark on similar efforts. .

## What are the main strengths and weaknesses?

The main strength of the CC-LAC is its regional scope and the large sample size. As discussed before (see ‘Why was the cohort set up?’ section), LAC has not participated in other global cohort data-pooling efforts to study cardiovascular risk factors and outcomes, leaving room to study cardio-metabolic risk estimates in this region. This work is therefore the first-ever cohort data-pooling study in LAC, which will ultimately produce solid evidence to guide clinical and public health practice in LAC. Previous data-pooling efforts in LAC have only focused on cross-sectional or survey data, and have shown relevant differences in relation to high-income countries.^36^^,^^37^ CC-LAC will complement and move these efforts to the next level, achieving larger impact in LAC healthcare practice, research and policy. Moreover, the fact that most of the pooled cohorts have begun in recent years is also a strength. They will provide evidence based on contemporary estimates, reflecting the present epidemiological scenario. The CC-LAC has managed to harmonize key cardio-metabolic risk factors that were measured following consistent protocols, e.g. repeated blood pressure measurements or standard laboratory methods for biomarkers. Lastly, with the large sample size, other relevant outcomes, such as diabetes risk, may also be investigated.

Data pooling comes with methodological challenges. Understanding the heterogeneity among cohorts in terms of population and sampling methodology, as well as variables collection and ascertainment, is relevant to find the best way to pool and harmonize available cohort data. Additional sources of heterogeneity, such as different levels of non-response rates, would be a limitation of any cohort data-pooling project. Future results should be interpreted considering all this and other limitations specific to each research question.

Like other cohort pooling studies, among the weaknesses we should point out the heterogeneity in the definition of cardiovascular events. Although pooled cohorts have followed adjudication processes, or these outcomes were based on death certificates, data were sometimes registered as 10th revision of the International Statistical Classification of Diseases and Related Health Problems (ICD-10) codes or in broader categories (e.g. cardiovascular deaths). This prevents us from studying some specific outcomes, such as haemorrhagic versus ischaemic stroke. This is not a criticism of our cohorts, but a call to health and vital registries authorities as well as science funders in LAC. Opportunities for additional follow-up rounds of established cohort studies, or to conduct follow-up rounds of large projects that may have been initiated as cross-sectional surveys could be supported. In this regard, for example, the PERU MIGRANT Study was established as a cross-sectional project,^38^ though with support from UPCH and intentional funders (e.g. GloCal Health Fellowship Program from the University of California Global Health Institute), the investigators managed to conduct two follow-up rounds.^39^ In Jamaica, the Jamaica Healthy Lifestyle Study, a repeated cross-sectional survey on non-communicable diseases risk factors has recently received National Institutes of Health (R01) funding to expand the cross-sectional work into a true longitudinal cohort.^40^ Finally, another limitation is the relative scarcity of data from the Caribbean. The CC-LAC will provide relevant information pooling all available cohorts from this region. We strongly believe that this cohort profile, and forthcoming publications, will allow us to identify additional sources from this region or will stimulate interest among researchers from the Caribbean to conduct local and multi-country cohort studies.

The lack of novel or more sophisticated cardio-metabolic risk factors may be a limitation as well; these could include inflammation markers (e.g. high-sensitive C-reactive protein) or lipid biomarkers (e.g. apolipoproteins). Some of the pooled cohort studies have, nevertheless, stored blood samples from their baseline rounds. We strongly believe that CC-LAC will enhance visibility of LAC cohort studies and promote regional collaboration. In the future, additional risk factors may be measured in some cohorts or new follow-up rounds may be conducted to overcome current limitations.

## Can I get hold of the data? Where can I find out more?

Data availability will be prioritized among CC-LAC members. New LAC cohort studies are welcomed to join the CC-LAC. Expressions of interest in additional collaborations will be received and handled by the CC-LAC steering committee. For further details on our work, please contact the corresponding author.

**Profile in a nutshell**

- We set up the Cohorts Consortium of Latin America and the Caribbean (CC-LAC) to fill knowledge gaps in cardiovascular medicine and prevention relevant to LAC. The CC-LAC is uniquely positioned to study long-term effects of cardio-metabolic risk factors and outcomes in LAC, providing relevant and strong local evidence as well as pragmatic tools to advance clinical medicine and public health in LAC.
- A total of 32 cohorts have been pooled, with an additional 7 cohorts that have not documented cardiovascular outcomes yet. Three of the 32 cohorts started before 1990 (6.4% of pooled sample), 9 in the 1990s (7.0%), 15 in the 2000s (80.5%) and 5 since 2010 (6.1%) for a total of 174 180 participants at baseline. Precisely 16.9% of the participants are men, and the mean age is 47.6 (46 in women and 55 in men) years.
- Pooled cohorts include baseline and one follow-up round. The mean follow-up time is 8.50 years (range: <1.0–27.7).
- We have pooled cardio-metabolic risk factors: anthropometrics, blood pressure, lipid and diabetes biomarkers, and non-fatal (stroke, myocardial infarction, revascularization) and fatal (stroke, myocardial infraction, revascularization, sudden death) cardiovascular outcomes.
- Currently, data are not available for collaborations outside the consortium.

## Cohorts Consortium of Latin America and the Caribbean (CC-LAC)

**Steering committee** (* equal contribution): Rodrigo M Carrillo-Larco (Imperial College London, UK); Mariachiara Di Cesare (Middlesex University, UK); Ian R Hambleton (The University of the West Indies, Barbados); Anselm Hennis (Pan American Health Organization, USA); Vilma Irazola (Institute for Clinical Effectiveness and Health Policy, Argentina); Dalia Stern* (National Institute of Public Health, Mexico); Catterina Ferreccio* (Pontificia Universidad Católica de Chile, Chile); Paulo Lotufo* (University of São Paulo, Brazil); Pablo Perel (London School of Hygiene and Tropical Medicine, UK); Edward W Gregg (Imperial College London, UK); Majid Ezzati (Imperial College London, UK); Goodarz Danaei (Harvard T.H. Chan School of Public Health, USA); J Jaime Miranda (Universidad Peruana Cayetano Heredia, Perú).

**Cohort collaborators** (* equal contribution; listed alphabetically by surname): Carlos A Aguilar-Salinas (Instituto Nacional de Ciencias Médicas y Nutrición, México)*; Ramón Alvarez-Váz (Universidad de la Republica, Uruguay)*; Marselle B Amadio (Centro Universitario Senac Santo Amaro, Brazil)*; Cecilia Baccino (Universidad de la Republica, Uruguay)*; Claudia Bambs (Pontificia Universidad Católica de Chile, Chile)*; João Luiz Bastos (Universidade Federal de Santa Catarina, Brazil)*; Gloria Beckles (Centers for Disease Control and Prevention , USA)*; Antonio Bernabe-Ortiz (Universidad Peruana Cayetano Heredia, Perú)*; Carla DO Bernardo (The University of Adelaide, Australia)*; Katia V Bloch (Universidade Federal do Rio de Janeiro, Brazil)*; Juan E Blümel (Universidad de Chile, Chile)*; Jose G Boggia (Universidad de la Republica, Uruguay)*; Pollyanna K Borges (Universidade Estadual de Ponta Grossa, Brazil)*; Miguel Bravo (MELISA Institute, Chile)*; Gilbert Brenes-Camacho (Universidad de Costa Rica, Costa Rica)*; Horacio A Carbajal (Universidad Nacional de la Plata, Argentina)*; Maria S Castillo Rascon (Universidad Nacional de Misiones, Argentina)*; Blanca H Ceballos (Hospital Dr Ramon Madariaga, Argentina)*; Veronica Colpani (Federal University of Rio Grande do Sul, Brazil)*; Susana C Confortin (Universidade Federal de Santa Catarina, Brazil)*; Jackie A Cooper (Queen Mary University of London, UK)*; Adrian Cortés-Valencia (National Institute of Public Health, Mexico)*; Sandra Cortes (Pontificia Universidad Católica de Chile, Chile)*; Roberto S Cunha (Federal University of Espírito Santo, Brazil)*; Eleonora d'Orsi (Universidade Federal de Santa Catarina, Brazil)*; William H Dow (University of California, Berkeley, USA)*; Walter G Espeche (Universidad Nacional de la Plata, Argentina)*; Flavio D Fuchs (Universidade Federal do Rio Grande do Sul, Brazil)*; Sandra C Fuchs (Universidade Federal do Rio Grande do Sul, Brazil)*; Suely GA Gimeno (Universidad Federal de São Paulo, Brazil)*; Donaji Gomez-Velasco (Instituto Nacional de Ciencias Médicas y Nutrición, México)*; Clicerio Gonzalez-Villalpando (Instituto Nacional de Salud Pública, México)*; María-Elena Gonzalez-Villalpando (Centro de Estudios en Diabetes A.C., México)*; David A Gonzalez-Chica (The University of Adelaide, Australia)*; Gonzalo Grazioli (Hospital Churruca Visca, Argentina)*; Ricardo O Guerra (Federal University of Rio Grande do Norte, Brazil)*; Laura Gutierrez (Institute for Clinical Effectiveness and Health Policy, Argentina)*; Fernando L Herkenhoff (Federal University of Espírito Santo, Brazil)*; Andrea RVR Horimoto (University of São Paulo, Brazil)*; Andrea Huidobro (Universidad Católica del Maule, Chile)*; Elard Koch (MELISA Institute, Chile)*; Martin Lajous (Harvard T.H. Chan School of Public Health, USA; National Institute of Public Health, Mexico)*; Maria Fernanda Lima-Costa (Oswaldo Cruz Foundation, Brazil)*; Ruy Lopez-Ridaura (National Institute of Public Health, Mexico)*; Alvaro CC Maciel (Federal University of Rio Grande do Norte, Brazil)*; Betty S Manrique-Espinoza (National Institute of Public Health, Mexico)*; Larissa P Marques (Universidade Federal de Santa Catarina, Brazil)*; Jose G Mill (Federal University of Espírito Santo, Brazil)*; Leila B Moreira (Universidade Federal do Rio Grande do Sul, Brazil)*; Lariane M Ono (Universidade Federal do Paraná, Brazil)*; Oscar M Muñoz (Pontificia Universidad Javeriana, Hospital Universitario San Ignacio, Colombia)*; Karen Oppermann (Passo Fundo University, Brazil)*; Sergio V Peixoto (Oswaldo Cruz Foundation, Brazil)*; Alexandre C Pereira (University of São Paulo, Brazil)*; Karen G Peres (Griffith University, Australia)*; Marco A Peres (Griffith University, Australia)*; Nohora I Rodriguez (Clinica de Marly, Colombia)*; Rosalba Rojas-Martinez (Instituto Nacional de Salud Pública, México)*; Luis Rosero-Bixby (Universidad de Costa Rica, Costa Rica)*; Adolfo Rubinstein (Institute for Clinical Effectiveness and Health Policy, Argentina)*; Alvaro Ruiz-Morales (Pontificia Universidad Javeriana, Colombia)*; Martin R Salazar (Universidad Nacional de la Plata, Argentina)*; Aaron Salinas-Rodriguez (National Institute of Public Health, Mexico)*; Ramon A Sanchez (Universidad Nacional de Misiones, Argentina)*; Ione JC Schneider (Universidade Federal de Santa Catarina, Brazil)*; Thiago LN Silva (Universidade Federal do Rio de Janeiro, Brazil)*; Nelson AS Silva (Universidade Federal do Rio de Janeiro, Brazil)*; Liam Smeeth (London School of Hygiene & Tropical Medicine, UK)*; Poli M Spritzer (Federal University of Rio Grande do Sul, Brazil)*; Fiorella Tartaglione (Hospital Churruca Visca, Argentina)*; Jorge Tartaglione (Hospital Churruca Visca, Argentina)*

## Supplementary data

Supplementary data are available at *IJE* online.

## Funding

Strategic Award, Wellcome Trust-Imperial College Centre for Global Health Research (100693/Z/12/Z). Imperial College London Wellcome Trust Institutional Strategic Support Fund [Global Health Clinical Research Training Fellowship] (294834/Z/16/Z ISSF ICL). R.C.-L. is supported by a Wellcome Trust International Training Fellowship (214185/Z/18/Z).

## Supplementary Material

dyaa073_supplementary_data
