# Erratum to: Cohort Profile: The Cohorts Consortium of Latin America and the Caribbean (CC-LAC) Cohorts Consortium of Latin America and the Caribbean (CC-LAC)

**DOI:** 10.1093/ije/dyab001

**Published:** 2021-02-20

First published online: 5 September 2020, *Int J Epidemiol* 2020;**49**:1437–1437g, doi: https://doi.org/10.1093/ije/dyaa073

When this article was originally published, the lists showing the Steering committee and Cohort collaborators for the Cohorts Consortium of Latin America and the Caribbean were published as supplementary material.

These lists now appear at the end of the article to allow proper attribution of authors and collaborators in the PubMed database.

