# Supplementary material for: Cohort Profile: The Cohorts Consortium of Latin America and the Caribbean (CC-LAC)
Source: Int J Epidemiol. 2020 Sep 5;49(5):1437–1437g. doi: 10.1093/ije/dyaa073 (PMC7746413; doi:10.1093/ije/dyaa073)
Supplement: dyaa073_supplementary_data [file dyaa073_supplementary_data.zip › CC-LAC Author list_AuthorsChecked.docx]

# **Cohorts Consortium of Latin America and the Caribbean (CC-LAC)**

**Steering committee** (* equal contribution): Rodrigo M Carrillo-Larco (Imperial College London, UK); Mariachiara Di Cesare (Middlesex University, UK); Ian R Hambleton (The University of the West Indies, Barbados); Anselm Hennis (Pan American Health Organization, USA); Vilma Irazola (Institute for Clinical Effectiveness and Health Policy, Argentina); Dalia Stern* (National Institute of Public Health, Mexico); Catterina Ferreccio* (Pontificia Universidad Católica de Chile, Chile); Paulo Lotufo* (University of São Paulo, Brazil); Pablo Perel (London School of Hygiene and Tropical Medicine, UK); Edward W Gregg (Imperial College London, UK); Majid Ezzati (Imperial College London, UK); Goodarz Danaei (Harvard T.H. Chan School of Public Health, USA); J Jaime Miranda (Universidad Peruana Cayetano Heredia, Perú).

**Cohort collaborators** (* equal contribution; listed alphabetically by surname): Carlos A Aguilar-Salinas (Instituto Nacional de Ciencias Médicas y Nutrición, México)*; Ramón Alvarez-Váz (Universidad de la Republica, Uruguay)*; Marselle B Amadio (Centro Universitario Senac Santo Amaro, Brazil)*; Cecilia Baccino (Universidad de la Republica, Uruguay)*; Claudia Bambs (Pontificia Universidad Católica de Chile, Chile)*; João Luiz Bastos (Universidade Federal de Santa Catarina, Brazil)*; Gloria Beckles (Centers for Disease Control and Prevention , USA)*; Antonio Bernabe-Ortiz (Universidad Peruana Cayetano Heredia, Perú)*; Carla DO Bernardo (The University of Adelaide, Australia)*; Katia V Bloch (Universidade Federal do Rio de Janeiro, Brazil)*; Juan E Blümel (Universidad de Chile, Chile)*; Jose G Boggia (Universidad de la Republica, Uruguay)*; Pollyanna K Borges (Universidade Estadual de Ponta Grossa, Brazil)*; Miguel Bravo (MELISA Institute, Chile)*; Gilbert Brenes-Camacho (Universidad de Costa Rica, Costa Rica)*; Horacio A Carbajal (Universidad Nacional de la Plata, Argentina)*; Maria S Castillo Rascon (Universidad Nacional de Misiones, Argentina)*; Blanca H Ceballos (Hospital Dr Ramon Madariaga, Argentina)*; Veronica Colpani (Federal University of Rio Grande do Sul, Brazil)*; Susana C Confortin (Universidade Federal de Santa Catarina, Brazil)*; Jackie A Cooper (Queen Mary University of London, UK)*; Adrian Cortés-Valencia (National Institute of Public Health, Mexico)*; Sandra Cortes (Pontificia Universidad Católica de Chile, Chile)*; Roberto S Cunha (Federal University of Espírito Santo, Brazil)*; Eleonora d'Orsi (Universidade Federal de Santa Catarina, Brazil)*; William H Dow (University of California, Berkeley, USA)*; Walter G Espeche (Universidad Nacional de la Plata, Argentina)*; Flavio D Fuchs (Universidade Federal do Rio Grande do Sul, Brazil)*; Sandra C Fuchs (Universidade Federal do Rio Grande do Sul, Brazil)*; Suely GA Gimeno (Universidad Federal de São Paulo, Brazil)*; Donaji Gomez-Velasco (Instituto Nacional de Ciencias Médicas y Nutrición, México )*; Clicerio Gonzalez-Villalpando (Instituto Nacional de Salud Pública, México)*; María-Elena Gonzalez-Villalpando (Centro de Estudios en Diabetes A.C., México)*; David A Gonzalez-Chica (The University of Adelaide, Australia)*; Gonzalo Grazioli (Hospital Churruca Visca, Argentina)*; Ricardo O Guerra (Federal University of Rio Grande do Norte, Brazil)*; Laura Gutierrez (Institute for Clinical Effectiveness and Health Policy, Argentina)*; Fernando L Herkenhoff (Federal University of Espírito Santo, Brazil)*; Andrea RVR Horimoto (University of São Paulo, Brazil)*; Andrea Huidobro (Universidad Católica del Maule, Chile)*; Elard Koch (MELISA Institute, Chile)*; Martin Lajous (Harvard T.H. Chan School of Public Health, USA; National Institute of Public Health, Mexico)*; Maria Fernanda Lima-Costa (Oswaldo Cruz Foundation, Brazil)*; Ruy Lopez-Ridaura (National Institute of Public Health, Mexico)*; Alvaro CC Maciel (Federal University of Rio Grande do Norte, Brazil)*; Betty S Manrique-Espinoza (National Institute of Public Health, Mexico)*; Larissa P Marques (Universidade Federal de Santa Catarina, Brazil)*; Jose G Mill (Federal University of Espírito Santo, Brazil)*; Leila B Moreira (Universidade Federal do Rio Grande do Sul, Brazil)*; Lariane M Ono (Universidade Federal do Paraná, Brazil)*; Oscar M Muñoz (Pontificia Universidad Javeriana, Hospital Universitario San Ignacio, Colombia)*; Karen Oppermann (Passo Fundo University, Brazil)*; Sergio V Peixoto (Oswaldo Cruz Foundation, Brazil)*; Alexandre C Pereira (University of São Paulo, Brazil)*; Karen G Peres (Griffith University, Australia)*; Marco A Peres (Griffith University, Australia)*; Nohora I Rodriguez (Clinica de Marly, Colombia)*; Rosalba Rojas-Martinez (Instituto Nacional de Salud Pública, México)*; Luis Rosero-Bixby (Universidad de Costa Rica, Costa Rica)*; Adolfo Rubinstein (Institute for Clinical Effectiveness and Health Policy, Argentina)*; Alvaro Ruiz-Morales (Pontificia Universidad Javeriana, Colombia)*; Martin R Salazar (Universidad Nacional de la Plata, Argentina)*; Aaron Salinas-Rodriguez (National Institute of Public Health, Mexico)*; Ramon A Sanchez (Universidad Nacional de Misiones, Argentina)*; Ione JC Schneider (Universidade Federal de Santa Catarina, Brazil)*; Thiago LN Silva (Universidade Federal do Rio de Janeiro, Brazil)*; Nelson AS Silva (Universidade Federal do Rio de Janeiro, Brazil)*; Liam Smeeth (London School of Hygiene & Tropical Medicine, UK)*; Poli M Spritzer (Federal University of Rio Grande do Sul, Brazil)*; Fiorella Tartaglione (Hospital Churruca Visca, Argentina)*; Jorge Tartaglione (Hospital Churruca Visca, Argentina)*
