# Supplementary material for: Cohort Profile: The Cohorts Consortium of Latin America and the Caribbean (CC-LAC)
Source: Int J Epidemiol. 2020 Sep 5;49(5):1437–1437g. doi: 10.1093/ije/dyaa073 (PMC7746413; doi:10.1093/ije/dyaa073)
Supplement: dyaa073_supplementary_data [file dyaa073_supplementary_data.zip › Supplementary Material IJE-2019-10-1345.R1_AuthorsChecked.docx]

**Supplementary Material**

**Cohort Profile: The Cohorts Consortium of Latin America and the Caribbean (CC-LAC)**

Cohorts Consortium of Latin America and the Caribbean (CC-LAC)

**Contents**

[**Terms used in the literature search to identify cohorts in Latin America and the Caribbean** 2](#_Toc38356626)

[**Reference(s) and/or website(s) that led to the collaborating cohorts** 3](#_Toc38356627)

[**Implausible values in selected cardio-metabolic risk factors** 7](#_Toc38356628)

[**Details about ascertainment of fatal cardiovascular events by cohort and sources of information to verify this methodology** 8](#_Toc38356629)

[**Details about ascertainment of non-fatal cardiovascular events by cohort and sources of information to verify this methodology** 13](#_Toc38356630)

[**Additional variables collated from collaborating cohorts** 17](#_Toc38356631)

# **Terms used in the literature search to identify cohorts in Latin America and the Caribbean**

| ("Antigua and Barbuda" OR "Argentina" OR "Bahamas" OR "Barbados" OR "Belize" OR "Bermuda" OR "Bolivia" OR "Brazil" OR "Chile" OR "Colombia" OR "Costa Rica" OR "Cuba" OR "Dominica" OR "Dominican Republic" OR "Ecuador" OR "El Salvador" OR "Grenada" OR "Guatemala" OR "Guyana" OR "Haiti" OR "Honduras" OR "Jamaica" OR "Mexico" OR "Nicaragua" OR "Panama" OR "Paraguay" OR "Peru" OR "Puerto Rico" OR "Saint Kitts and Nevis" OR "Saint Vincent and the Grenadines" OR "Saint Lucia" OR "Suriname" OR "Trinidad and Tobago" OR "Uruguay" OR "Venezuela") |
| --- |

| ("Follow-Up Studies" OR "Longitudinal Studies" OR "Prospective Studies" OR Cohort stud* OR Prospective cohort* OR Prospective stud*) |
| --- |

| ("Stroke" OR "Cardiovascular Diseases" OR Cardiovascular diseas* OR Cardiovascular event* OR stroke* OR brain-vascular accident* OR CVA OR cerebrovascular accident* OR heart attack* OR myocardial infarction* OR "Myocardial Infarction") |
| --- |

These three groups were connected with the Boolean operator “AND”.

# **Reference(s) and/or website(s) that led to the collaborating cohorts**

| **Cohort (country)** | **Years of data collection** | **Contact source** |
| --- | --- | --- |
| Costa Rican Longevity and Healthy Aging Study – CRELES | 2005, 2007, 2009 | Rosero-Bixby L, Coto-Yglesias F, Dow WH. Pulse blood pressure and cardiovascular mortality in a population-based cohort of elderly Costa Ricans. J Hum Hypertens. 2016;30(9):555-62.  <http://www.creles.berkeley.edu/> |
| Puerto Rico Heart Health Program (PRHHP) | 1965, 1980 | <https://biolincc.nhlbi.nih.gov/studies/prhhp/> |
| Rauch City (Argentina) | 1997,  2003, 2012 | Salazar MR, Espeche WG, Aizpurúa M, Leiva Sisnieguez BC, Balbín E, Dulbecco CA, Carbajal HA. Blood pressure response to a community-based program and long-term cardiovascular outcome. Am J Hypertens. 2014;27(8):1061-8. |
| St James Survey (Trinidad & Tobago) | 1977, 1986 | Miller GJ, Cooper JA, Beckles GL. Cardiorespiratory fitness, all-cause mortality, and risk of cardiovascular disease in Trinidadian men--the St James survey. Int J Epidemiol. 2005;34(6):1387-94. |
| 10/66 Dementia Study (Cuba, Dominican Republic, Peru, Venezuela, Mexico, Puerto Rico) | 2003, 2013  (varies for each country) | Prina AM, Acosta D, Acosta I, Guerra M, Huang Y, Jotheeswaran AT, Jimenez-Velazquez IZ, Liu Z, Llibre Rodriguez JJ, Salas A, Sosa AL, Williams JD, Prince M. Cohort Profile: The 10/66 study. Int J Epidemiol. 2017;46(2):406-406i.  Prince M, Ferri CP, Acosta D, Albanese E, Arizaga R, Dewey M, Gavrilova SI, Guerra M, Huang Y, Jacob KS, Krishnamoorthy ES, McKeigue P, Rodriguez JL, Salas A, Sosa AL, Sousa RM, Stewart R, Uwakwe R. The protocols for the 10/66 dementia research group population-based research programme. BMC Public Health. 2007;7:165.  <https://www.alz.co.uk/1066/> |
| Validación de los modelos de predicción de Framingham y PROCAM como estimadores del riesgo cardiovascular en una población colombiana (Colombia) | 1984-1994, 1996-2006 | Oscar M. Muñoz, Nohora I. Rodríguez, Álvaro Ruiz, Martín Rondón, Validación de los modelos de predicción de Framingham y PROCAM como estimadores del riesgo cardiovascular en una población colombiana, Revista Colombiana de Cardiología. 2014;21(4):202-212. |
| The Bambuí (Brazil) Cohort Study of Ageing | 1997, 2011 | Lima-Costa MF, Firmo JO, Uchoa E. Cohort profile: the Bambui (Brazil) Cohort Study of Ageing. Int J Epidemiol. 2011;40(4):862-7. |
| Eventos cardiovasculares en una población cerrada (Argentina) | 1997, 2006 | Tartaglione, J, Grazioli, GC, Sarmiento, M, Goldstraj, LM. Eventos cardiovasculares en una población cerrada. Seguimiento a 10 años. Revista Argentina de Cardiología. 2008;76(5):347-351. |
| Anthropometric Indexes Predicting Cardiometabolic Risk. Prospective Cohort Study in a Population of Employees of Public Hospitals (Argentina) | 2001, 2012 | Sánchez A, Muhn MA, Lovera M, Ceballos B, Bonneau G, Pedrozo W et al . Índices antropométricos predicen riesgo cardiometabólico: Estudio de cohorte prospectivo en una población de empleados de hospitales públicos. Rev. argent. endocrinol. metab. 2014;51(4):185-191. |
| Estudio Barros Luco | 1997, 2018 | Castelo-Branco C, Blümel JE, Roncagliolo ME, Haya J, Bolf D, Binfa L, Tacla X, Colodrón M. Age, menopause and hormone replacement therapy influences on cardiovascular risk factors in a cohort of middle-aged Chilean women. Maturitas. 2003;45(3):205-12. |
| GEnotipo, Fenotipo y Ambiente de la HiperTensión Arterial en UruguaY – GEFA-HT-UY (Uruguay) | 2012, 2016 | Luzardo Leonella, Lujambio Inés, Sottolano Mariana, Da Rosa Alicia, Robaina Sebastián, Arce Federico et al . Cohorte GEFA-HT-UY (GEnotipo, Fenotipo y Ambiente de la HiperTensión Arterial en UruguaY): Protocolo y primeros resultados. Rev. Méd. Urug. 2013;29(2):103-113. |
| Japanese-Brazilian Diabetes Study Group (JBDSG) | 1993, 2007 | Bevilacqua Marselle Rodrigues, Gimeno Suely Godoy Agostinho. Abdominal obesity in Japanese-Brazilians: which measure is best for predicting all-cause and cardiovascular mortality? Cad. Saúde Pública. 2011;27(10):1986-1996. |
| CESCAS (Argentina, Chile, Uruguay) | 2010, 2018 | Rubinstein AL, Irazola VE, Poggio R, Bazzano L, Calandrelli M, Lanas Zanetti FT, Manfredi JA, Olivera H, Seron P, Ponzo J, He J. Detection and follow-up of cardiovascular disease and risk factors in the Southern Cone of Latin America: the CESCAS I study. BMJ Open. 2011;1(1):e000126. |
| The Mexican Teachers' Cohort (MTC) | 2006-2008, 2011-2014,  2014-2019 | Lajous M, Ortiz-Panozo E, Monge A, Santoyo-Vistrain R, García-Anaya A, Yunes-Díaz E, Rice MS, Blanco M, Hernández-Ávila M, Willett WC, Romieu I, López-Ridaura R. Cohort Profile: The Mexican Teachers' Cohort (MTC). Int J Epidemiol. 2017;46(2):e10. |
| The Mexico City Diabetes Study | 1991, 2009 | González-Villalpando C, Dávila-Cervantes CA, Zamora-Macorra M, Trejo-Valdivia B, González-Villalpando ME. Incidence of type 2 diabetes in Mexico: results of the Mexico City Diabetes Study after 18 years of follow-up. Salud Publica Mex. 2014;56(1):11-7. |
| Porto Alegre, Brazil – Cohort | 1990, 1997 | Moraes RS, Fuchs FD, Moreira LB, Wiehe M, Pereira GM, Fuchs SC. Risk factors for cardiovascular disease in a Brazilian population-based cohort study. Int J Cardiol. 2003;90(2-3):205-11. |
| The Passo Fundo Cohort Study (Brazil) | 1995, 2001, 2011 | Oppermann K, Colpani V, Fuchs SC, Spritzer PM. The Passo Fundo Cohort Study: design of a population-based observational study of women in premenopause, menopausal transition, and postmenopause. Women's Midlife Health. 2015;1:12  Colpani V, Oppermann K, Spritzer PM. Causes of death and associated risk factors among climacteric women from Southern Brazil: a population based-study. BMC Public Health. 2014;14:194. |
| Study on global AGEing and adult health (SAGE) – Mexico | 2009, 2014 | Kowal P, Chatterji S, Naidoo N, Biritwum R, Fan W, Lopez Ridaura R, Maximova T, Arokiasamy P, Phaswana-Mafuya N, Williams S, Snodgrass JJ, Minicuci N, D'Este C, Peltzer K, Boerma JT; SAGE Collaborators. Data resource profile: the World Health Organization Study on global AGEing and adult health (SAGE). Int J Epidemiol. 2012;41(6):1639-49. |
| EpiFloripa Ageing Study (Brazil) | 2009,  2013,  2017 | <http://epifloripa.paginas.ufsc.br/>  Schneider, I., Confortin, S., Bernardo, C., Bolsoni, C., Antes, D., Pereira, K., Ono, L., Marques, L., Borges, L., Giehl, M., Krug, R., Goes, V., Boing, A., Boing, A. F., & d’Orsi, E.. EpiFloripa Aging cohort study: methods, operational aspects, and follow-up strategies. Revista De Saúde Pública. 2017;51:104. |
| Maule Cohort (MAUCO) of chronic diseases, Chile | 2014, 2017 | Ferreccio C, Roa JC, Bambs C, Vives A, Corvalán AH, Cortés S, Foerster C, Acevedo J, Huidobro A, Passi A, Toro P, Covacevich Y, de la Cruz R, Koshiol J, Olivares M, Miquel JF, Cruz F, Silva R, Quest AF, Kogan MJ, Castro PF, Lavandero S. Study protocol for the Maule Cohort (MAUCO) of chronic diseases, Chile 2014-2024. BMC Public Health. 2016;16:122. |
| Costa Rican Longevity and Healthy Aging Study – CRELES 1945-1955 Retirement Cohort | 2010, 2012 | <http://www.creles.berkeley.edu/> |
| CRONICAS Cohort Study (Peru) | 2010, 2012, 2013 | Miranda JJ, Bernabe-Ortiz A, Smeeth L, Gilman RH, Checkley W; CRONICAS Cohort Study Group. Addressing geographical variation in the progression of non-communicable diseases in Peru: the CRONICAS cohort study protocol. BMJ Open. 2012;2(1):e000610. |
| PERU MIGRANT Study (Peru) | 2007, 2012, 2015 | Carrillo-Larco RM, Ruiz-Alejos A, Bernabé-Ortiz A, Gilman RH, Smeeth L, Miranda JJ. Cohort Profile: The PERU MIGRANT Study-A prospective cohort study of rural dwellers, urban dwellers and rural-to-urban migrants in Peru. Int J Epidemiol. 2017;46(6):1752-1752f. |
| Mexican Health & Aging Study (MHAS) | 2001, 2003, 2012, 2015 | <http://www.mhasweb.org/> |
| Impact of cognitive deficit on survival among elderly residents in the community (Brazil) | 2002, 2007 | Campos Cavalcanti Maciel A, Oliveira Guerra R, Villaverde Gutiérrez C. [Impact of cognitive deficit on survival among elderly residents in the community]. Rev Esp Geriatr Gerontol. 2008;43(6):337-45. |
| Puerto Rican Elderly: Health Conditions (PREHCO) | 2002, 2006 | <http://prehco.rcm.upr.edu/> |
| Baependi Heart Study (Brazil) | 2005, 2010 | Egan KJ, von Schantz M, Negrão AB, Santos HC, Horimoto ARVR, et al. Cohort profile: the Baependi Heart Study—a family-based, highly admixed cohort study in a rural Brazilian town. BMJ Open. 2016;6:e011598. |
| St Francisco Project (Chile) | 1997, 2006 | Koch Elard, Bravo Miguel, Romero Camila, Diaz Aldo, Castañeda Héctor, Aguilera Hernán et al . Estatura, factores de riesgo cardiovascular y riesgo de mortalidad en adultos: Proyecto San Francisco, Chile. Rev Chil Cardiol. 2011;30(3):198-206. |
| EpiFloripa Adults Cohort Study | 2009, 2012, 2014 | Peres Marco Aurelio, Peres Karen Glazer, Boing Antonio Fernando, Bastos João Luiz, Silva Diego Augusto, González-Chica David Alejandro. Oral health in the EpiFloripa: a prospective study of adult health in Southern Brazil. Rev. bras. epidemiol. 2014( 2 ): 571-575.  Boing Alexandra Crispim, Peres Karen Glazer, Boing Antonio Fernando, Hallal Pedro C, Silva Nilza Nunes, Peres Marco Aurélio. EpiFloripa Health Survey: the methodological and operational aspects behind the scenes. Rev. bras. epidemiol. 2014. 17( 1 ): 147-162. |
| Metabolic Syndrome Cohort | 2006, 2014 | Arellano-Campos O, Gómez-Velasco DV, Bello-Chavolla OY, Cruz-Bautista I, Melgarejo-Hernandez MA, Muñoz-Hernandez L, Guillén LE, Garduño-Garcia JJ, Alvirde U, Ono-Yoshikawa Y, Choza-Romero R, Sauque-Reyna L, Garay-Sevilla ME, Malacara-Hernandez JM, Tusie-Luna MT, Gutierrez-Robledo LM, Gómez-Pérez FJ, Rojas R, Aguilar-Salinas CA. Development and validation of a predictive model for incident type 2 diabetes in middle-aged Mexican adults: the metabolic syndrome  cohort. BMC Endocr Disord. 2019;19(1):41. |
| MONICA-VITÓRIA | 1999, 2004 | Pereira AC, Mota GF, Cunha RS, Herbenhoff FL, Mill JG, Krieger JE. Angiotensinogen 235T allele "dosage" is associated with blood pressure phenotypes. Hypertension. 2003;41(1):25-30. |
| Hipertensão Arterial na Ilha do Governador - HAIG-Long (Hypertension in Ilha do Governador) | 1991, 2009 | da Silva TL, Klein CH, Nogueira Ada R, Salis LH, de Souza E Silva NA, Bloch KV. Cardiovascular mortality among a cohort of hypertensive and normotensives in Rio de Janeiro - Brazil - 1991-2009. BMC Public Health. 2015;15:623. |

In the pooled database, a cohort unit is the cohort-country, i.e. multi-country cohort studies are counted as many times as countries they have (e.g., CESCAS-Argentina, CESCAS-Chile, CESCAS-Uruguay; CESCAS is one unique cohort but three cohort units). There are 32 unique cohort studies listed above (25 pooled and 7 not pooled yet), they represent 32 pooled cohort units and 7 cohort units not pooled yet. In total the CC-LAC is a consortium of 39 cohorts (32 pooled and 7 not pooled yet).

# **Implausible values in selected cardio-metabolic risk factors**

Implausible values in the selected risk factors were re-coded to missing. Overall pooled sample size is 174,180.

| **Risk Factor** | **Implausible value criteria** | **N (re-coded to missing)** |
| --- | --- | --- |
| Systolic blood pressure | < 70 mmHg | 12 |
|  | > 270 mmHg | 1 |
| Diastolic blood pressure | < 30 mmHg | 8 |
|  | > 150 mmHg | 7 |
| Total cholesterol | < 67.77 mg/dL (1.75 mmol/L) | 9 |
|  | > 773.3 mg/dL (>20.00 mmol/L) | 1 |
| HDL cholesterol | < 15.46 mg/dL (<0.40 mmol/L) | 60 |
|  | > 193.3 mg/dL (>5.00 mmol/L) | 0 |
| Body mass index | < 10 Kg/m^2^ | 2 |
|  | > 80 Kg/m^2^ | 0 |
| Glucose | < 45.05 mg/dL (<2.50 mmol/L) | 37 |
|  | > 540.54 mg/dL (>30.00 mmol/L) | 4 |

# **Details about ascertainment of fatal cardiovascular events by cohort and sources of information to verify this methodology**

| **Cohort** | **Deaths** | **References** |
| --- | --- | --- |
| CRELES | Survival status in CRELES was established in two ways: (1) through the computer records in the National Death Registry up to December 31, 2010, and (2) during the second (2006-2008) and third (2008-2010) waves of home visits. The computer follow-up used the unique identification number (the cédula) that all Costa Ricans have. Five out of the 566 deaths found in the field were not found in the Registry, suggesting a death under-registration rate of 1%. In contrast, 10% of the deaths from the Registry were not found in the field, appearing in the second and third waves as loss of follow-up. For the foreigners in the sample (~3%), survival was established only in the field because they did not have a unique identification number with which to link them to the Registry. Record linkage with the vital statistics databases, provided by the National Statistics and Census Institute (INEC), allowed us to identify the basic cause of death for 96% of the deceased. In conclusion: the ample used in this cohort followed a questionnaire-based approach to ascertain deaths (case and dates); most of these deaths were found (thus confirmed) in national death registries. The PI provided the ICD-10 causes of death; these were not available online. | <https://www.demographic-research.org/volumes/vol30/7/30-7.pdf> |
|  |  | <https://pophealthmetrics.biomedcentral.com/track/pdf/10.1186/1478-7954-10-11> |
|  |  | <https://www.demographic-research.org/volumes/vol38/3/38-3.pdf> |
|  |  | <https://www.nature.com/articles/jhh2015117.pdf> |
| CRELES Retirement | There was an “exit questionnaire” for target subjects and spouses who were interviewed during the first wave but died before they were interviewed at second wave. Interviewers had to interview a proxy respondent who must have been close to the deceased subject and who had enough information about the subject’s last days. What was the primary disease that lead to the death of (NAME)? In conclusion: no specific details about cross-checking with national registries; death details were asked in a questionnaire. The PI confirmed they did not cross-check with national registries. | <http://www.creles.berkeley.edu/pdf/Methods_RC_w2.pdf> |
|  |  | <http://www.creles.berkeley.edu/pdf/DeceasedRC_w2.pdf> |
| CRONICAS Cohort Study | In the household, a next-of-kin was asked whether the participant was alive or has died since the last visit; if s/he has died, then questions about when and cause were asked (at the first follow-up, cause of death was not recorded). If available, a copy of the death certificate was requested to record these details. In conclusion: questions to a next-of-kin and when available, death certificates; no further validation process. | Provided by the collaborators |
| PERU MIGRANT Study | Retrieved from national death registries, based on death certificates. In conclusion: death information from national death registries (death certificates). | Provided by the collaborators |
| MHAS | Information retrieved through paper-based questionnaires applied to the next-of-kin (i.e., someone in close and frequent contact with the study subject); questionnaire applied to a *substitute informant*. In conclusion: death information was retrieved from questionnaires applied to an informant (next-of-kin) without further verification. | <http://www.mhasweb.org/DiscussionForum/ViewTopic.aspx?Topic=90&Forum=33&MHAS=0&ForumName=&TopicSubject=&Views=0> |
| PRHHP | Adjudicated. Medical history was elicited. The review panel was composed of a minimum of three study physicians including two cardiologists, the study pathologist, the statistician and the medical records librarian. The diagnosis, including cause of death, was determined by this review group always following the previously established criteria that applied to all study participants. At regular intervals, staff associated with the Framingham Heart Study participated in case review sessions. In conclusion: adjudication based on medical history by a physician panel. | <https://academic.oup.com/ije/article-lookup/doi/10.1093/ije/dyv150> |
|  |  | <https://www.sciencedirect.com/science/article/pii/0895435688901333> |
| Rauch City | A structured interview was conducted among participants by specially trained nurses and social workers. The collected data were then evaluated by a highly qualified internist (blinded with respect to the subject’s baseline cardiovascular risk factors) to assign a specific outcome to every event. When necessary, available medical records were also reviewed. Information based on verbal autopsy and interviews with conclusions adjudicated by a physician (internist). In conclusion: based on verbal autopsy and interview with next-of-kin, and when relevant, available medical records were reviewed; events were adjudicated by a physician. | <https://academic.oup.com/ajh/article-lookup/doi/10.1093/ajh/hpt240> |
| St James Survey | Non-fatal events and underlying cause of death were arrived at after the review of all documentation (including hospital notes, necropsy records, laboratory reports, and history from the subject and family) by two physicians, with a third as arbiter when required. In conclusion: events adjudicated by two physicians based on medical and death records as well as information from relatives. | <https://academic.oup.com/ije/article/34/6/1387/707488> |
| PREHCO | Questionnaire applied to a proxy asking about the causes and circumstances of death. In conclusion: questions applied to a next-of-kin about details of the death; no further verification with other sources. | <http://prehco.rcm.upr.edu/> |
| 10/66 Dementia Study | For those who died between baseline and follow-up (information ascertained on follow-up visit) we completed a verbal autopsy interview with a co-resident, relative or other person well-placed to know the circumstances of death, using methods developed and validated by the 'Million Deaths' project for use in India to identify underlying cause according to ICD10 criteria. Cause of death is allocated by the consensus judgement of two physicians. The approach is valid for use up to three years post-mortem. In conclusion: based on a verbal autopsy applied to a relative, causes were adjudicated by physicians. | <https://bmcpublichealth.biomedcentral.com/articles/10.1186/1471-2458-7-165> |
| Validación de los modelos de predicción de Framingham y PROCAM como estimadores del riesgo cardiovascular en una población colombiana | Regarding deaths, cause was determined based on the death certificate or discharge note. In conclusion: fatal cases were verified with deaths certificates. | <http://www.scielo.org.co/pdf/rcca/v21n4/v21n4a02.pdf> |
| The Bambuí (Brazil) Cohort Study of Ageing | Annual follow-up visits including verification of death certificates. In conclusion: death information based on death certificates. | <https://academic.oup.com/ije/article/40/4/862/678230> |
| Eventos cardiovasculares en una población cerrada | Study in a captive population: policemen. All events were verified with clinical records at the only hospital for this population. Deaths that occurred outside this hospital were verified with deaths registries (*base de datos de la obra social*). In conclusion: fatal events were verified with clinical records or death registries. | <http://www.redalyc.org/articulo.oa?id=305327059005> |
| Impact of cognitive deficit on survival among elderly residents in the community | Death information was collected from relatives or local death registries. In conclusion: fatal events were retrieved from local death registries or provided by relatives. | <http://www.elsevier.es/es-revista-revista-espanola-geriatria-gerontologia-124-pdf-S0211139X08751886-S300> |
| Anthropometric Indexes Predicting Cardiometabolic Risk. Prospective Cohort Study in a Population of Employees of Public Hospitals | Collaborators reported that these events were verified with death registries based on death certificates. In conclusion: deaths were verified with death registries based on death certificates. | <http://www.scielo.org.ar/scielo.php?script=sci_arttext&pid=S1851-30342014000400003> |
| Estudio Barros Luco | In 2017, using the national identification number, the national death records were reviewed, consigning for each participating woman, their vital status (alive or death), date and cause of death. In conclusion: death information was retrieved from deaths records based on death certificates. | <https://www.maturitas.org/article/S0378-5122(03)00140-3/pdf> |
| GEFA-HT-UY | Death information was retrieved from interviews with relatives and verified with clinical records or death certificates. This was confirmed by collaborators by email. In conclusion: death events were provided by relatives and verified with written sources. | <http://www.rmu.org.uy/revista/29/2/2/es/5/> |
| JBDSG | Collaborators reported that these events were identified with death certificates; latest publication reports that it was possible to identify the date and cause of death based on a death certificate in 90.6% of cases. In conclusion: death information was based on death certificates. | <http://www.scielo.br/scielo.php?script=sci_arttext&pid=S0102-311X2011001000012> |
| CESCAS | The study outcome committee reviewed the collected source documents related to the reported events and adjudicated all CVD outcomes independently. The collaborators reported: death certificates, verbal autopsies and interviews. In conclusion: fatal events were based on death certificates or adjudicated. | <http://ppct.caicyt.gov.ar/index.php/rac/article/view/12908> |
|  |  | <https://bmjopen.bmj.com/content/1/1/e000126> |
| MTC | They identified deaths through Teachers’ Incentives Program (TIP), Social Security and Services for Civil Servants (ISSSTE), next-of-kin call centre interviews and the postal service. Using the Mexican Death Registry, they established the cause of death. In conclusion: death events were based on death certificates. | <https://academic.oup.com/ije/article/46/2/e10/3038108> |
| The Mexico City Diabetes Study | The collaborators reported: death certificates, verbal autopsies and adjudication as well as simple interviews. In conclusion: death events were based on death certificates and adjudicated. | <https://www.scielosp.org/scielo.php?script=sci_arttext&pid=S0036-36342014000100003&lng=en&nrm=iso&tlng=en> |
| Porto Alegre, Brazil – Cohort | The collaborator reported: death certificates, verbal autopsy (adjudication) and simple interviews. Also: the events were adjudicated using a collection of paper forms obtained from verbal necropsy, hospital charts, death certificate. The cause of death was determined as well as the date and entered the data set. As soon as we calculated the time to the event, those dates were deleted. The final decision regarding the cause of death took in consideration hospital information and interview with relatives. Two researchers adjudicated the causes, and these were verified by a third researcher. In conclusion: death events were based on death certificates and adjudicated. | <https://www.ncbi.nlm.nih.gov/pubmed/12957753> |
| The Passo Fundo Cohort Study | All 358 participants or their relatives were reached, and information regarding participant deaths was obtained for the period ending in November 2011. In addition to the interviews, the medical records of city hospitals and the Center for Health Information (NIS/RS-SES) were reviewed. All deaths between 1995 and 2011 were included. Medical records were reviewed to collect information on age at death, date, and cause of death. The causes of death were coded using the International Classification of Diseases, 10th revision. In conclusion: death events were based on clinical records. | https://www.ncbi.nlm.nih.gov/pubmed/24559309 |
| Epifloripa Cohort Study of Ageing (Brazil) | Fatal events verified with registries based on death certificates. In conclusion: mortality information verified with valid sources (e.g., death certificates) | Provided by the collaborators |
| SAGE – Mexico | Cause of death not yet processed, though collected following the WHO verbal autopsy methodology. In conclusion: not available yet, mortality information being processed. | Provided by the collaborators |
| St Francisco Project (Chile) | Cause of death verified with death certificates. In conclusion: mortality information verified with valid sources (death certificates) | Provided by the collaborators |
| Baependi Heart Study (Brazil) | No cause of death available currently. In conclusion: mortality information is not yet available (currently being collected). | Provided by the collaborators |
| Maule Cohort (MAUCO) of chronic diseases, Chile | Collaborators reported that deaths were verified with death certificates for cause and date of death. In conclusion: fatal events were based on death certificates. | Provided by the collaborators |
| EpiFloripa Adults Cohort Study | Collaborators reported that deaths were verified with death certificates. In conclusion: fatal events were based on death certificates | Provided by the collaborators |
| Metabolic Syndrome Cohort | Collaborators confirmed that fatal events were verified with written sources such as clinical records. In conclusion: fatal events were verified with written sources. | Provided by the collaborators |
| MONICA-VITÓRIA | No hard endpoints collected. In conclusion: fatal events are not available. | Provided by the collaborators |
| Hipertensão Arterial na Ilha do Governador - HAIG-Long (Hypertension in Ilha do Governador) | Collaborators confirmed that fatal events were verified with written sources such as death certificates. In conclusion: fatal events were verified with written sources. | Provided by the collaborators |

# **Details about ascertainment of non-fatal cardiovascular events by cohort and sources of information to verify this methodology**

| **Cohort** | **Non-Fatal Events** | **References** |
| --- | --- | --- |
| CRELES | No specific information or papers retrieved. *During the last 2 years has a physician told you that you have had a heart attack (yes, no, DK, NR)?* & *How many heart attacks have you had in the last two years (#)?* & *How long ago did you have each of the attacks (less than a year, more than a year, DK)?* Same questions for stroke. Same questions in 2007 and 2009. In conclusion: stroke and myocardial infarction (MI) retrieved with questionnaires. | <http://www.creles.berkeley.edu/documentation.html> |
| CRELES Retirement | No specific information or papers retrieved. *During the last 2 years has a doctor told you that you have had an infarct or heart attack (yes, no, DK, NR)?* & *How many infarcts of attacks have you had in the last two years (#)?* Same questions for stroke. In conclusion: stroke and MI retrieved with questionnaires. | <http://www.creles.berkeley.edu/documentation.html> |
| CRONICAS Cohort Study | *During the last 12 months, have you presented with some of the following health problems [heart attack and stroke listed] (yes, no, DK)?* Month and year of diagnosis were recorded as well. These questions available in both follow-up rounds. In conclusion: based on questionnaires; month and year of the event (self-reported) available. | Provided by the collaborators |
| PERU MIGRANT Study | *Since the first time you were interviewed (5 years ago), have you ever had one of the following health problems [infarction or heart attack and stroke listed] (yes, no, DK)?* Month and year of diagnosis were recorded too. In conclusion: based on questionnaires; month and year of the event (self-reported) available. | Provided by the collaborators |
| MHAS | In 2003: *Doctor ever say you had a heart attack (yes, no)? When have last heart attack (year)?* Same questions for stroke. In 2012: *Has a physician ever told respondent...heart attack (yes, no, RF, DK)?* then, *respondent's year of most recent heart attack (year)?* for stroke, *Ever/last 2 years has a physician told respondent...stroke (yes, no, RF, DK?* and then, *respondent's year of recent stroke.* 2015 questionnaire is not available yet, though the corresponding variables (same name) have the same structure (thus likely similar questions were posed). In conclusion: stroke and MI ascertained with questionnaires; year (self-reported) available. | <http://www.mhasweb.org/DocumentationQuestionnaire.aspx> |
| PRHHP | Adjudicated by a panel of physicians. Medical history was elicited. Only MI available. In conclusion: MI adjudicated based on medical history by a physician panel; no stroke data, | <https://academic.oup.com/ije/article-lookup/doi/10.1093/ije/dyv150> |
|  |  | <https://www.sciencedirect.com/science/article/pii/0895435688901333> |
| Rauch City | A structured interview was conducted among participants by specially trained nurses and social workers. The collected data were then evaluated by a highly qualified internist to assign a specific outcome to every event. When necessary, available medical records were also reviewed. In conclusion: MI and stroke were adjudicated by a physician based on questionnaires and medical records (when necessary and available). | <https://academic.oup.com/ajh/article-lookup/doi/10.1093/ajh/hpt240> |
|  |  |  |
| St James Survey | Non-fatal events and underlying cause of death were arrived at after the review of all documentation (including hospital notes, necropsy records, laboratory reports, and history from the subject and family) by two physicians, with a third as arbiter when required. In conclusion: stroke and MI were adjudicated by two physicians based on medical records as well as information from relatives. | <https://academic.oup.com/ije/article/34/6/1387/707488> |
| PREHCO | *Since the last interview, have you had any heart attack (yes, no, DK)? How old were you at the latest heart attack?* Same questions for stroke. In conclusion: stroke and MI were retrieved with questionnaires; age at the time of the event available. | <http://prehco.rcm.upr.edu/> |
| 10/66 Dementia Study | Question about MI diagnosed by a physician available; though no exact date is provided. However, they ask for "heart problems" (heart attack could be single out) and when the participant was told s/he had this problem (0-5 years ago, 5-10 years ago, 11+ years ago). The also included a two-phase clinical protocol designed to identify incident stroke. All those claiming (by participant or informant report) to have experienced a stroke in the interval between assessments, and all those with suggestive neurological signs not apparent at baseline were offered physician assessment including physical examination, clinical history, and examination of clinical notes and investigations where available. We sought consensus diagnosis from two local independent experts; however, no time information is available. In conclusion: stroke was adjudicated by a physician while MI was based on questionnaires of self-reported diagnosis and who made the diagnosis; however, the collaborator confirmed this was not the case and therefore (strong) non-fatal events are not available. | <https://bmcpublichealth.biomedcentral.com/articles/10.1186/1471-2458-7-165> |
| Validación de los modelos de predicción de Framingham y PROCAM como estimadores del riesgo cardiovascular en una población colombiana | Medical records of subjects who had a cardiovascular event were assessed by an independent expert (specialist in Internal Medicine) who confirmed the event. In conclusion: non-fatal events were verified with medical records by a medical specialist. | <http://www.scielo.org.co/pdf/rcca/v21n4/v21n4a02.pdf> |
| The Bambuí (Brazil) Cohort Study of Ageing | Information not collected. In conclusion: no information about non-fatal events. | <https://academic.oup.com/ije/article/40/4/862/678230> |
| Eventos cardiovasculares en una población cerrada | Study in a captive population: policemen. All events were verified with clinical records at the only hospital for this population. In conclusion: non-fatal events were verified with clinical records. | <http://www.redalyc.org/articulo.oa?id=305327059005> |
| Impact of cognitive deficit on survival among elderly residents in the community | They only recorded stroke information; no information available for MI. In addition, they only recorded the occurrence of the event, not the exact time. The diagnostic was verified with a written source. In conclusion: collaborators reported non-fatal stroke was confirmed with a written source such as medical records. | <http://www.elsevier.es/es-revista-revista-espanola-geriatria-gerontologia-124-pdf-S0211139X08751886-S300> |
| Anthropometric Indexes Predicting Cardiometabolic Risk. Prospective Cohort Study in a Population of Employees of Public Hospitals | Collaborators reported that these events were verified with written sources such as clinical records. In conclusion: non-fatal events were verified with written sources such as clinical records. | <http://www.scielo.org.ar/scielo.php?script=sci_arttext&pid=S1851-30342014000400003> |
| Estudio Barros Luco | Up to June 2018, non-fatal events and dates were updated with a national compulsory health record. In conclusion: non-fatal events were retrieved from a national compulsory record of non-communicable events. | <https://www.maturitas.org/article/S0378-5122(03)00140-3/pdf> |
| GEFA-HT-UY | Events information was retrieved from interviews with relatives and verified with clinical records or other documents from the health provider. This was confirmed by collaborators by email. In conclusion: non-fatal events were provided by relatives and verified with written sources (e.g., clinical records). | <http://www.rmu.org.uy/revista/29/2/2/es/5/> |
| JBDSG | Information not collected. In conclusion: no information about non-fatal events. | <http://www.scielo.br/scielo.php?script=sci_arttext&pid=S0102-311X2011001000012> |
| CESCAS | The study outcome committee reviewed the collected source documents related to the reported events and adjudicate all CVD outcomes independently. The collaborators reported: death certificates, verbal autopsies and interviews. In conclusion: non-fatal events were adjudicated. | <http://ppct.caicyt.gov.ar/index.php/rac/article/view/12908> |
|  |  | <https://bmjopen.bmj.com/content/1/1/e000126> |
| MTC | Information not collected. In conclusion: no information about non-fatal events. | <https://academic.oup.com/ije/article/46/2/e10/3038108> |
| The Mexico City Diabetes Study | EKGs were available (for MI), but no information on date of the even was provided hence this outcome was not included. In conclusion: no information about non-fatal events. | <https://www.scielosp.org/scielo.php?script=sci_arttext&pid=S0036-36342014000100003&lng=en&nrm=iso&tlng=en> |
| Porto Alegre, Brazil – Cohort | The collaborator reported: The events were adjudicated using a collection of paper forms obtained from verbal necropsy, hospital charts, death certificates. The events were determined as well as the date and entered the data set. As soon as we calculated the time to the event, those dates were deleted. In conclusion: non-fatal events were adjudicated based on several documents. | <https://www.ncbi.nlm.nih.gov/pubmed/12957753> |
| The Passo Fundo Cohort Study | The collaborator reported that the non-fatal events were reported by the participants. In conclusion: non-fatal events were not verified with written sources. | https://www.ncbi.nlm.nih.gov/pubmed/24559309 |
|  |  | <https://womensmidlifehealthjournal.biomedcentral.com/articles/10.1186/s40695-015-0013-8> |
| Baependi Heart Study (Brazil) | Records not available. In conclusion: non-fatal events are not available. | Provided by the collaborators |
| SAGE – Mexico | Non-fatal events based on self-reports alone. In conclusion: non-fatal events were not verified with written sources. | Provided by the collaborators |
| St Francisco Project (Chile) | Incidence of non-fatal events were made by a physician or in a health facility, and verified with written sources (e.g., clinical records). In conclusion: non-fatal events verified with clinical records. | Provided by the collaborators |
| Epifloripa Cohort Study of Ageing (Brazil) | Records not available. In conclusion: non-fatal events are not available. | Provided by the collaborators |
| Maule Cohort (MAUCO) of chronic diseases, Chile | Collaborators confirmed that non-fatal events were verified with written sources such as clinical records. In conclusion: non-fatal events were verified with written sources. | Provided by the collaborators |
| EpiFloripa Adults Cohort Study | Non-fatal events not available yet. In conclusion: no information available. | Provided by the collaborators |
| Metabolic Syndrome Cohort | Collaborators confirmed that non-fatal events were verified with written sources such as clinical records. In conclusion: non-fatal events were verified with written sources. | Provided by the collaborators |
| MONICA-VITÓRIA | No hard endpoints collected. In conclusion: non-fatal events are not available. | Provided by the collaborators |
| Hipertensão Arterial na Ilha do Governador - HAIG-Long | Collaborators confirmed that non-fatal events were not collected. In conclusion: non-fatal events are not available. | Provided by the collaborators |

# **Additional variables collated from collaborating cohorts**

The black cells represent if the variables are available, the white (with 0) cells show when the variables are not available.

| **Variable** | **CRONICAS** | **PERUMIGRANT** | **MHAS** | **PREHCO** | **CRELES** | **CRELES Retirement** | **Rauch City** | **PRHHP** | **St James Survey** | **Validación de los modelos de predicción de …** | **The Bambuí (Brazil) Cohort Study** | **Eventos cardiovasculares en una población cerrada...** | **Impact of cognitive deficit on survival…** | **Anthropometric Indexes Predicting** | **Estudio Barros Luco** | **GEFA-HT-UY** | **10/66 Dementia Study** | **JBDSG** | **CESCAS** | **MTC** | **The Mexico City Diabetes Study** | **Porto Alegre, Brazil – Cohort** | **Passo Fundo Cohort** | **Epifloripa Cohort Study of Ageing** | **Baependi Heart Study** | **St Francisco Project** | **SAGE – Mexico** | **MAUCO** | **EpiFloripa Adults Cohort Study** | **Metabolic Syndrome Cohort** | **MONICA-VITÓRIA** | **HAIG-Long** |
| --- | --- | --- | --- | --- | --- | --- | --- | --- | --- | --- | --- | --- | --- | --- | --- | --- | --- | --- | --- | --- | --- | --- | --- | --- | --- | --- | --- | --- | --- | --- | --- | --- |
| Baseline age | 1 | 1 | 1 | 1 | 1 | 1 | 1 | 1 | 1 | 1 | 1 | 1 | 1 | 1 | 1 | 1 | 1 | 1 | 1 | 1 | 1 | 1 | 1 | 1 | 1 | 1 | 1 | 1 | 1 |  | 1 | 1 |
| Sex | 1 | 1 | 1 | 1 | 1 | 1 | 1 | 1 | 1 | 1 | 1 | 1 | 1 | 1 | 1 | 1 | 1 | 1 | 1 | 1 | 1 | 1 | 1 | 1 | 1 | 1 | 1 | 1 | 1 |  | 1 | 1 |
| Location (urban/rural) | 1 | 1 | 1 | 1 | 1 | 1 | 1 | 1 | 1 | 0 | 1 | 1 | 1 | 1 | 1 | 1 | 1 | 1 | 1 | 1 | 1 | 1 | 1 | 1 | 1 | 1 | 1 | 0 | 0 |  | 1 | 1 |
| Education (years) | 1 | 1 | 1 | 1 | 1 | 1 | 1 | 1 | 0 | 0 | 1 | 0 | 1 | 1 | 0 | 1 | 1 | 1 | 1 | 0 | 1 | 1 | 1 | 1 | 1 | 1 | 1 | 1 | 1 |  | 1 | 0 |
| Hypertension diagnosis | 1 | 1 | 1 | 1 | 1 | 1 | 1 | 0 | 1 | 0 | 1 | 1 | 1 | 1 | 1 | 1 | 1 | 0 | 1 | 1 | 1 | 1 | 1 | 1 | 1 | 1 | 1 | 1 | 1 |  | 1 | 1 |
| Hypertension drug | 1 | 1 | 1 | 1 | 0 | 1 | 1 | 1 | 0 | 0 | 1 | 1 | 0 | 1 | 0 | 1 | 1 | 1 | 1 | 1 | 1 | 1 | 0 | 0 | 1 | 1 | 1 | 1 | 1 |  | 1 | 1 |
| Diabetes diagnosis | 1 | 1 | 1 | 1 | 1 | 1 | 0 | 1 | 1 | 1 | 1 | 1 | 1 | 1 | 1 | 1 | 1 | 0 | 1 | 1 | 1 | 1 | 1 | 1 | 1 | 1 | 1 | 1 | 1 |  | 1 | 1 |
| Diabetes drug | 1 | 1 | 1 | 1 | 1 | 1 | 1 | 1 | 0 | 0 | 1 | 0 | 0 | 0 | 0 | 1 | 1 | 1 | 1 | 1 | 1 | 0 | 0 | 0 | 1 | 1 | 1 | 1 | 1 |  | 1 | 1 |
| Alcohol consumption variable | 1 | 1 | 1 | 1 | 1 | 1 | 1 | 0 | 1 | 0 | 1 | 0 | 0 | 0 | 1 | 1 | 1 | 1 | 1 | 1 | 1 | 1 | 1 | 1 | 1 | 1 | 1 | 1 | 1 |  | 0 | 1 |
| Alcohol consumption variable 1 | 1 | 1 | 1 | 1 | 1 | 1 | 1 | 0 | 0 | 0 | 0 | 0 | 0 | 0 | 0 | 0 | 0 | 1 | 0 | 0 | 1 | 0 | 1 | 1 | 0 | 0 | 0 | 0 | 1 |  | 0 | 0 |
| Alcohol consumption variable 2 | 1 | 1 | 1 | 0 | 1 | 0 | 0 | 0 | 0 | 0 | 0 | 0 | 0 | 0 | 0 | 0 | 0 | 1 | 0 | 0 | 1 | 0 | 0 | 1 | 0 | 0 | 0 | 0 | 0 | 0 | 0 | 0 |
| Alcohol consumption variable 3 | 0 | 0 | 1 | 0 | 0 | 0 | 0 | 0 | 0 | 0 | 0 | 0 | 0 | 0 | 0 | 0 | 0 | 1 | 0 | 0 | 0 | 0 | 0 | 0 | 0 | 0 | 0 | 0 | 0 | 0 | 0 | 0 |
| Current smoker | 1 | 1 | 1 | 1 | 1 | 1 | 1 | 1 | 1 | 1 | 1 | 1 | 0 | 1 | 1 | 1 | 1 | 1 | 1 | 1 | 1 | 1 | 1 | 1 | 1 | 1 | 1 | 1 | 1 |  | 1 | 1 |
| Current smoker 1 | 1 | 1 | 1 | 1 | 1 | 1 | 1 | 1 | 0 | 0 | 0 | 0 | 0 | 0 | 0 | 0 | 0 | 1 | 0 | 0 | 1 | 0 | 1 | 1 | 0 | 0 | 0 | 0 | 0 |  | 1 | 0 |
| Current smoker 2 | 1 | 1 | 1 | 0 | 1 | 0 | 0 | 0 | 0 | 0 | 0 | 0 | 0 | 0 | 0 | 0 | 0 | 1 | 0 | 0 | 1 | 0 | 0 | 1 | 0 | 0 | 0 | 0 | 1 | 0 | 0 | 0 |
| Current smokers 3 | 0 | 0 | 1 | 0 | 0 | 0 | 0 | 0 | 0 | 0 | 0 | 0 | 0 | 0 | 0 | 0 | 0 | 1 | 0 | 0 | 0 | 0 | 0 | 0 | 0 | 0 | 0 | 0 | 0 | 0 | 0 | 0 |
| Systolic blood pressure | 1 | 1 | 0 | 0 | 1 | 1 | 1 | 1 | 1 | 1 | 1 | 1 | 0 | 1 | 1 | 1 | 1 | 1 | 1 | 1 | 1 | 1 | 1 | 1 | 1 | 1 | 1 | 1 | 1 |  | 1 | 1 |
| Systolic blood pressure 1 | 1 | 1 | 0 | 0 | 1 | 1 | 1 | 1 | 0 | 0 | 1 | 0 | 0 | 0 | 0 | 0 | 0 | 1 | 0 | 0 | 1 | 0 | 1 | 1 | 0 | 0 | 0 | 1 | 1 |  | 1 | 0 |
| Systolic blood pressure 2 | 1 | 1 | 1 | 0 | 1 | 0 | 0 | 1 | 0 | 0 | 1 | 0 | 0 | 0 | 0 | 0 | 0 | 1 | 0 | 0 | 1 | 0 | 0 | 0 | 0 | 0 | 0 | 0 | 1 | 0 | 0 | 0 |
| Diastolic blood pressure | 1 | 1 | 0 | 0 | 1 | 1 | 1 | 1 | 1 | 1 | 1 | 1 | 0 | 1 | 1 | 1 | 1 | 1 | 1 | 1 | 1 | 1 | 1 | 1 | 1 | 1 | 1 | 1 | 1 |  | 1 | 1 |
| Diastolic blood pressure 1 | 1 | 1 | 0 | 0 | 1 | 1 | 1 | 1 | 0 | 0 | 0 | 0 | 0 | 0 | 0 | 0 | 0 | 1 | 0 | 0 | 1 | 0 | 1 | 1 | 0 | 0 | 0 | 1 | 1 |  | 1 | 0 |
| Diastolic blood pressure 2 | 1 | 1 | 1 | 0 | 1 | 0 | 0 | 1 | 0 | 0 | 0 | 0 | 0 | 0 | 0 | 0 | 0 | 1 | 0 | 0 | 1 | 0 | 0 | 0 | 0 | 0 | 0 | 0 | 1 | 0 | 0 | 0 |
| Weight | 1 | 1 | 1 | 1 | 1 | 1 | 1 | 1 | 1 | 1 | 1 | 1 | 0 | 1 | 1 | 1 | 0 | 1 | 1 | 1 | 1 | 1 | 1 | 1 | 1 | 1 | 1 | 1 | 1 |  | 1 | 1 |
| Weight 1 | 1 | 1 | 1 | 1 | 1 | 1 | 1 | 1 | 0 | 0 | 1 | 0 | 0 | 0 | 0 | 0 | 0 | 1 | 0 | 0 | 1 | 0 | 1 | 1 | 0 | 0 | 0 | 0 | 0 |  | 1 | 0 |
| Weight 2 | 1 | 1 | 1 | 0 | 1 | 0 | 0 | 1 | 0 | 0 | 1 | 0 | 0 | 0 | 0 | 0 | 0 | 1 | 0 | 0 | 1 | 0 | 0 | 1 | 0 | 0 | 0 | 0 | 1 | 0 | 0 | 0 |
| Height | 1 | 1 | 1 | 1 | 1 | 1 | 1 | 1 | 1 | 1 | 1 | 1 | 0 | 1 | 1 | 1 | 1 | 1 | 1 | 1 | 1 | 1 | 0 | 1 | 1 | 1 | 1 | 1 | 1 |  | 1 | 1 |
| Height 1 | 1 | 1 | 1 | 1 | 1 | 0 | 0 | 1 | 0 | 0 | 1 | 0 | 0 | 0 | 0 | 0 | 0 | 1 | 0 | 0 | 1 | 0 | 1 | 1 | 0 | 0 | 0 | 0 | 0 |  | 1 | 0 |
| Height 2 | 0 | 0 | 1 | 0 | 1 | 0 | 0 | 0 | 0 | 0 | 1 | 0 | 0 | 0 | 0 | 0 | 0 | 1 | 0 | 0 | 1 | 0 | 1 | 1 | 0 | 0 | 0 | 0 | 1 | 0 | 0 | 0 |
| Waist | 1 | 1 | 1 | 1 | 1 | 1 | 1 | 0 | 0 | 0 | 1 | 0 | 0 | 1 | 0 | 1 | 1 | 1 | 1 | 1 | 1 | 1 | 1 | 1 | 1 | 1 | 1 | 0 | 1 |  | 1 | 0 |
| Waist 1 | 1 | 1 | 1 | 1 | 1 | 1 | 1 | 0 | 0 | 0 | 1 | 0 | 0 | 0 | 0 | 0 | 0 | 1 | 0 | 0 | 1 | 0 | 1 | 1 | 0 | 0 | 0 | 1 | 0 |  | 1 | 0 |
| Waist 2 | 1 | 0 | 1 | 0 | 1 | 0 | 0 | 0 | 0 | 0 | 1 | 0 | 0 | 0 | 0 | 0 | 0 | 1 | 0 | 0 | 1 | 0 | 0 | 1 | 0 | 0 | 0 | 0 | 1 | 0 | 0 | 0 |
| Hip | 1 | 1 | 1 | 1 | 1 | 1 | 1 | 0 | 0 | 0 | 1 | 0 | 0 | 0 | 0 | 1 | 1 | 1 | 0 | 1 | 1 | 1 | 1 | 0 | 1 | 0 | 1 | 0 | 0 |  | 1 | 0 |
| Hip 1 | 0 | 1 | 1 | 1 | 1 | 1 | 0 | 0 | 0 | 0 | 1 | 0 | 0 | 0 | 0 | 0 | 0 | 1 | 0 | 0 | 1 | 0 | 1 | 0 | 0 | 0 | 0 | 1 | 0 |  | 1 | 0 |
| Hip 2 | 1 | 0 | 1 | 0 | 1 | 0 | 0 | 0 | 0 | 0 | 1 | 0 | 0 | 0 | 0 | 0 | 0 | 1 | 0 | 0 | 1 | 0 | 0 | 0 | 0 | 0 | 0 | 0 | 0 | 0 | 0 | 0 |
| BMI | 1 | 1 | 1 | 1 | 1 | 1 | 1 | 1 | 1 | 1 | 1 | 1 | 0 | 0 | 1 | 1 | 0 | 1 | 1 | 1 | 1 | 1 | 1 | 1 | 1 | 1 | 1 | 1 | 1 |  | 1 | 1 |
| BMI 1 | 1 | 1 | 1 | 1 | 1 | 1 | 1 | 1 | 0 | 0 | 1 | 0 | 0 | 0 | 0 | 0 | 0 | 1 | 0 | 0 | 1 | 0 | 1 | 1 | 0 | 0 | 0 | 0 | 0 |  | 1 | 0 |
| BMI 2 | 1 | 1 | 1 | 0 | 1 | 0 | 0 | 1 | 0 | 0 | 1 | 0 | 0 | 0 | 0 | 0 | 0 | 1 | 0 | 0 | 1 | 0 | 0 | 1 | 0 | 0 | 0 | 0 | 1 | 0 | 0 | 0 |
| Skinfold triceps | 0 | 1 | 0 | 0 | 1 | 1 | 0 | 1 | 1 | 0 | 1 | 0 | 0 | 0 | 0 | 1 | 0 | 0 | 0 | 0 | 1 | 0 | 1 | 0 | 0 | 0 | 0 | 0 | 0 | 0 | 1 | 0 |
| Skinfold triceps 1 | 0 | 0 | 0 | 0 | 1 | 1 | 0 | 1 | 0 | 0 | 1 | 0 | 0 | 0 | 0 | 0 | 0 | 0 | 0 | 0 | 1 | 0 | 0 | 0 | 0 | 0 | 0 | 0 | 0 | 0 | 1 | 0 |
| Skinfold triceps 2 | 0 | 0 | 0 | 0 | 1 | 0 | 0 | 0 | 0 | 0 | 1 | 0 | 0 | 0 | 0 | 0 | 0 | 0 | 0 | 0 | 1 | 0 | 0 | 0 | 0 | 0 | 0 | 0 | 0 | 0 | 0 | 0 |
| Skinfold subscapular | 0 | 1 | 0 | 0 | 1 | 1 | 0 | 0 | 1 | 0 | 0 | 0 | 0 | 0 | 0 | 1 | 0 | 0 | 0 | 0 | 1 | 0 | 1 | 0 | 0 | 0 | 0 | 0 | 0 | 0 | 0 | 0 |
| Skinfold subscapular 1 | 0 | 0 | 0 | 0 | 1 | 1 | 0 | 1 | 0 | 0 | 0 | 0 | 0 | 0 | 0 | 0 | 0 | 0 | 0 | 0 | 1 | 0 | 0 | 0 | 0 | 0 | 0 | 0 | 0 | 0 | 0 | 0 |
| Skinfold subscapular 2 | 0 | 0 | 0 | 0 | 1 | 0 | 0 | 0 | 0 | 0 | 0 | 0 | 0 | 0 | 0 | 0 | 0 | 0 | 0 | 0 | 1 | 0 | 0 | 0 | 0 | 0 | 0 | 0 | 0 | 0 | 0 | 0 |
| Skinfold biceps | 0 | 1 | 0 | 0 | 0 | 0 | 0 | 0 | 1 | 0 | 0 | 0 | 0 | 0 | 0 | 1 | 0 | 0 | 0 | 0 | 0 | 0 | 0 | 0 | 0 | 0 | 0 | 0 | 0 | 0 | 0 | 0 |
| Skinfold supra | 0 | 1 | 0 | 0 | 0 | 0 | 0 | 0 | 0 | 0 | 0 | 0 | 0 | 0 | 0 | 0 | 0 | 0 | 0 | 0 | 0 | 0 | 1 | 0 | 0 | 0 | 0 | 0 | 0 | 0 | 0 | 0 |
| Total cholesterol | 1 | 1 | 0 | 0 | 1 | 1 | 1 | 1 | 1 | 1 | 1 | 1 | 0 | 1 | 1 | 1 | 1 | 1 | 1 | 1 | 1 | 0 | 1 | 1 | 1 | 1 | 0 | 1 | 0 |  | 1 | 0 |
| Total cholesterol 1 | 0 | 0 | 0 | 0 | 1 | 0 | 1 | 1 | 0 | 0 | 1 | 0 | 0 | 0 | 0 | 0 | 0 | 1 | 0 | 0 | 1 | 0 | 1 | 0 | 0 | 0 | 0 | 0 | 0 |  | 1 | 0 |
| Total cholesterol 2 | 1 | 0 | 1 | 0 | 0 | 0 | 0 | 1 | 0 | 0 | 0 | 0 | 0 | 0 | 0 | 0 | 0 | 1 | 0 | 0 | 1 | 0 | 0 | 0 | 0 | 0 | 0 | 0 | 1 | 0 | 0 | 0 |
| HDL cholesterol | 1 | 1 | 0 | 0 | 1 | 1 | 0 | 0 | 1 | 1 | 1 | 1 | 0 | 1 | 1 | 1 | 1 | 1 | 1 | 1 | 1 | 0 | 1 | 1 | 1 | 1 | 0 | 1 | 0 |  | 1 | 0 |
| HDL cholesterol 1 | 0 | 0 | 0 | 0 | 1 | 0 | 1 | 0 | 0 | 0 | 1 | 0 | 0 | 0 | 0 | 0 | 0 | 1 | 0 | 0 | 1 | 0 | 1 | 0 | 0 | 0 | 0 | 0 | 0 |  | 1 | 0 |
| HDL cholesterol 2 | 1 | 0 | 1 | 0 | 0 | 0 | 0 | 0 | 0 | 0 | 0 | 0 | 0 | 0 | 0 | 0 | 0 | 1 | 0 | 0 | 1 | 0 | 0 | 0 | 0 | 0 | 0 | 0 | 1 | 0 | 0 | 0 |
| LDL cholesterol | 1 | 1 | 0 | 0 | 1 | 0 | 0 | 0 | 1 | 1 | 1 | 1 | 0 | 1 | 1 | 1 | 1 | 1 | 1 | 1 | 1 | 0 | 1 | 1 | 1 | 1 | 0 | 1 | 0 |  | 1 | 0 |
| LDL cholesterol 1 | 0 | 0 | 0 | 0 | 1 | 0 | 0 | 0 | 0 | 0 | 1 | 0 | 0 | 0 | 0 | 0 | 0 | 1 | 0 | 0 | 0 | 0 | 1 | 0 | 0 | 0 | 0 | 0 | 0 |  | 1 | 0 |
| LDL cholesterol 2 | 1 | 0 | 0 | 0 | 0 | 0 | 0 | 0 | 0 | 0 | 0 | 0 | 0 | 0 | 0 | 0 | 0 | 1 | 0 | 0 | 0 | 0 | 0 | 0 | 0 | 0 | 0 | 0 | 1 | 0 | 0 | 0 |
| Triglycerides | 1 | 1 | 0 | 0 | 1 | 0 | 0 | 0 | 1 | 1 | 1 | 1 | 0 | 1 | 1 | 1 | 1 | 1 | 1 | 1 | 1 | 0 | 1 | 1 | 1 | 1 | 0 | 1 | 0 |  | 1 | 0 |
| Triglycerides 1 | 0 | 0 | 0 | 0 | 1 | 0 | 1 | 1 | 0 | 0 | 1 | 0 | 0 | 0 | 0 | 0 | 0 | 1 | 0 | 0 | 1 | 0 | 1 | 0 | 0 | 0 | 0 | 0 | 0 |  | 1 | 0 |
| Triglyceride 2 | 1 | 0 | 0 | 0 | 0 | 0 | 0 | 0 | 0 | 0 | 0 | 0 | 0 | 0 | 0 | 0 | 0 | 1 | 0 | 0 | 1 | 0 | 0 | 0 | 0 | 0 | 0 | 0 | 1 | 0 | 0 | 0 |
| High-sensitive C-reactive protein | 1 | 1 | 0 | 0 | 1 | 1 | 0 | 0 | 0 | 0 | 1 | 0 | 0 | 0 | 0 | 0 | 0 | 0 | 0 | 0 | 1 | 0 | 0 | 1 | 0 | 0 | 0 | 0 | 0 |  | 0 | 0 |
| High-sensitive C-reactive protein 1 | 0 | 0 | 0 | 0 | 1 | 0 | 0 | 0 | 0 | 0 | 0 | 0 | 0 | 0 | 0 | 0 | 0 | 0 | 0 | 0 | 0 | 0 | 0 | 0 | 0 | 0 | 0 | 0 | 0 |  | 0 | 0 |
| High-sensitive C-reactive protein 2 | 1 | 0 | 1 | 0 | 0 | 0 | 0 | 0 | 0 | 0 | 0 | 0 | 0 | 0 | 0 | 0 | 0 | 0 | 0 | 0 | 1 | 0 | 0 | 0 | 0 | 0 | 0 | 0 | 0 | 0 | 0 | 0 |
| Glucose | 1 | 1 | 0 | 0 | 1 | 0 | 1 | 1 | 1 | 0 | 1 | 1 | 0 | 1 | 1 | 1 | 1 | 1 | 1 | 1 | 1 | 0 | 1 | 1 | 1 | 1 | 0 | 1 | 0 |  | 1 | 0 |
| Glucose 1 | 0 | 0 | 0 | 0 | 1 | 0 | 1 | 1 | 0 | 0 | 1 | 0 | 0 | 0 | 0 | 0 | 0 | 1 | 0 | 0 | 1 | 0 | 1 | 0 | 0 | 0 | 0 | 0 | 0 |  | 1 | 0 |
| Glucose 2 | 1 | 1 | 0 | 0 | 0 | 0 | 0 | 1 | 0 | 0 | 0 | 0 | 0 | 0 | 0 | 0 | 0 | 1 | 0 | 0 | 1 | 0 | 0 | 0 | 0 | 0 | 0 | 0 | 1 | 0 | 0 | 0 |
| HbA1c | 1 | 1 | 0 | 0 | 1 | 1 | 0 | 0 | 1 | 0 | 0 | 0 | 0 | 0 | 0 | 0 | 0 | 0 | 0 | 0 | 0 | 0 | 0 | 1 | 1 | 0 | 0 | 0 |  | 0 | 0 | 0 |
| HbA1c 1 | 0 | 0 | 0 | 0 | 1 | 0 | 0 | 0 | 0 | 0 | 0 | 0 | 0 | 0 | 0 | 0 | 0 | 0 | 0 | 0 | 0 | 0 | 0 | 0 | 0 | 0 | 0 | 0 | 0 | 0 | 0 | 0 |
| HbA1c 2 | 1 | 0 | 0 | 0 | 0 | 0 | 0 | 0 | 0 | 0 | 0 | 0 | 0 | 0 | 0 | 0 | 0 | 0 | 0 | 0 | 0 | 0 | 0 | 0 | 0 | 0 | 0 | 0 | 0 | 0 | 0 | 0 |
| HbA1c 3 | 0 | 0 | 0 | 0 | 0 | 0 | 0 | 0 | 0 | 0 | 0 | 0 | 0 | 0 | 0 | 0 | 0 | 0 | 0 | 0 | 1 | 0 | 0 | 0 | 0 | 0 | 0 | 0 | 0 | 0 | 0 | 0 |
| Creatinine (any) | 1 | 0 | 0 | 0 | 0 | 0 | 0 | 0 | 0 | 0 | 1 | 0 | 0 | 0 | 0 | 1 | 1 | 0 | 1 | 0 | 1 | 0 | 0 | 0 | 1 | 0 | 0 | 0 | 0 | 0 | 1 | 0 |
| TSH 2 | 0 | 0 | 1 | 0 | 0 | 0 | 0 | 0 | 0 | 0 | 0 | 0 | 0 | 0 | 0 | 0 | 0 | 0 | 0 | 0 | 0 | 0 | 0 | 0 | 0 | 0 | 0 | 0 | 0 |  | 0 | 0 |
| TSH 4 | 0 | 0 | 0 | 0 | 0 | 0 | 0 | 0 | 0 | 0 | 0 | 0 | 0 | 0 | 0 | 0 | 0 | 0 | 0 | 0 | 1 | 0 | 0 | 0 | 0 | 0 | 0 | 0 | 0 | 0 | 0 | 0 |
| Vitamin D 2 | 0 | 0 | 1 | 0 | 0 | 0 | 0 | 0 | 0 | 0 | 0 | 0 | 0 | 0 | 0 | 0 | 0 | 0 | 0 | 0 | 0 | 0 | 0 | 0 | 0 | 0 | 0 | 0 | 0 | 0 | 0 | 0 |
| Fibrinogen | 0 | 1 | 0 | 0 | 0 | 0 | 0 | 0 | 0 | 0 | 0 | 0 | 0 | 0 | 0 | 0 | 0 | 0 | 0 | 0 | 0 | 0 | 0 | 0 | 0 | 0 | 0 | 0 | 0 | 0 | 0 | 0 |
| BMI was computed from measured weight and height, except for one cohort (Mexico Teachers' Cohort). The number as suffix at the end of each variable name represents follow-up rounds (e.g., 1 = first follow-up), not number of available measurements in each round; in other words, whether these are available at baseline, follow-up 1, follow-up 2, and so forth. Black shade where this variable is available, white (with 0) when it is NOT available. | | | | | | | | | | | | | | | | | | | | | | | | | | | | | | | | |
